# *HEATR2* Plays a Conserved Role in Assembly of the Ciliary Motile Apparatus

**DOI:** 10.1371/journal.pgen.1004577

**Published:** 2014-09-18

**Authors:** Christine P. Diggle, Daniel J. Moore, Girish Mali, Petra zur Lage, Aouatef Ait-Lounis, Miriam Schmidts, Amelia Shoemark, Amaya Garcia Munoz, Mihail R. Halachev, Philippe Gautier, Patricia L. Yeyati, David T. Bonthron, Ian M. Carr, Bruce Hayward, Alexander F. Markham, Jilly E. Hope, Alex von Kriegsheim, Hannah M. Mitchison, Ian J. Jackson, Bénédicte Durand, Walter Reith, Eamonn Sheridan, Andrew P. Jarman, Pleasantine Mill

**Affiliations:** 1 School of Medicine, University of Leeds, Leeds, United Kingdom; 2 Centre for Integrative Physiology, School of Biomedical Sciences, University of Edinburgh, Edinburgh, United Kingdom; 3 MRC Human Genetics Unit, Institute of Genetics and Molecular Medicine at The University of Edinburgh, Western General Hospital, Edinburgh, United Kingdom; 4 Department of Pathology and Immunology, Faculty of Medicine, Université de Genève, Geneva, Switzerland; 5 Molecular Medicine Unit and Birth Defect Research Center, Institute of Child Health, University College London, London, United Kingdom; 6 Paediatric Respiratory Department, Royal Brompton Hospital, London, United Kingdom; 7 Systems Biology Ireland, University College Dublin, Belfield, Dublin, Ireland; 8 Centre de Génétique et de Physiologie Moléculaire et Cellulaire, UMR 5534 CNRS, Université Claude Bernard Lyon 1, Villeurbanne, France; Washington University School of Medicine, United States of America

## Abstract

Cilia are highly conserved microtubule-based structures that perform a variety of sensory and motility functions during development and adult homeostasis. In humans, defects specifically affecting motile cilia lead to chronic airway infections, infertility and laterality defects in the genetically heterogeneous disorder Primary Ciliary Dyskinesia (PCD). Using the comparatively simple *Drosophila* system, in which mechanosensory neurons possess modified motile cilia, we employed a recently elucidated cilia transcriptional RFX-FOX code to identify novel PCD candidate genes. Here, we report characterization of *CG31320/HEATR2*, which plays a conserved critical role in forming the axonemal dynein arms required for ciliary motility in both flies and humans. Inner and outer arm dyneins are absent from axonemes of *CG31320* mutant flies and from PCD individuals with a novel splice-acceptor *HEATR2* mutation. Functional conservation of closely arranged RFX-FOX binding sites upstream of *HEATR2* orthologues may drive higher cytoplasmic expression of HEATR2 during early motile ciliogenesis. Immunoprecipitation reveals HEATR2 interacts with DNAI2, but not HSP70 or HSP90, distinguishing it from the client/chaperone functions described for other cytoplasmic proteins required for dynein arm assembly such as DNAAF1-4. These data implicate *CG31320/HEATR2* in a growing intracellular pre-assembly and transport network that is necessary to deliver functional dynein machinery to the ciliary compartment for integration into the motile axoneme.

## Author Summary

Cilia are small, specialized projections extending from a cell's surface that play key sensory and sometimes motility functions, such as generating fluid flow for clearing airways or sperm propulsion necessary for male fertility. Ciliary motility is defective in the inherited disease, Primary Ciliary Dyskinesia (PCD). Although the basic cilium blueprint has been elaborated on during evolution, many of the core genes involved in building or maintaining functional cilia have been conserved. We have used the comparatively simple fruit fly, which has motile cilia on only a handful of touch-sensitive sensory cells, to identify genes involved in ciliary motility and which are therefore candidate genes for causing PCD. We show here that when one such gene (*CG31320/HEATR2*) is disrupted in either flies or in human PCD patients, cilia form but they cannot move. We show this protein stays in the cytoplasm, where it is acts like a flexible scaffold stabilizing and facilitating interactions during the assembly of large multi-component ciliary motor complexes needed to power cilia movement.

## Introduction

Cilia and flagella are small microtubule-based projections on the cell surface, where they perform diverse sensory, and in some cases motility functions. These highly conserved organelles are found across species from protozoa to mammals, and are believed to have evolved from flagellar structures found in the last eukaryotic common ancestor (LECA) [1]. At their core is an axoneme composed of a peripheral arrangement of 9 microtubule doublets. Extension and maintenance of cilia involves a conserved microtubule-based process of motor-driven intraflagellar transport (IFT) that traffics protein cargo from the ciliary base to the tip, and back again. Despite the functional and structural diversity that has arisen among cilia [2], they retain key elements. Axonemes of motile cilia, which usually have an additional central pair of singlet microtubules in a “9+2” arrangement, possess inner (IDA) and outer dynein arms (ODA) attached to the peripheral outer doublet A microtubule. These orchestrate the ATP-dependent sliding of the doublets relative to each other, enabling motility. Recent genomic and proteomic studies have compiled a “motile ciliome”, now consisting of several hundred centrosomal and ciliary components. However, how these components are assembled into different structural and functional cilia types remain largely unknown.

Disorders specifically arising from dysfunction of motile cilia are called Primary Ciliary Dyskinesias (PCD, MIM244400). As a result of defective airway mucociliary clearance, individuals with PCD typically present in the first year of life with recurrent infections, rhinosinusitis and otitis media, resulting in a chronic respiratory condition which can progress to permanent lung damage (bronchiectasis). Around half of PCD patients also have laterality defects as a result of embryonic nodal cilia dysfunction that lead to randomization of the left-right body axis, most commonly situs inversus totalis (Kartagener syndrome) or more rarely heterotaxy defects affecting the heart. Fertility defects are also reported in individuals with PCD. PCD is genetically heterogeneous, and 27 causative loci have been identified to date, but these still account for only a fraction of total cases [3]–[6]. Diagnosis of PCD typically involves identification of ultrastructural defects of the motile ciliary axoneme, over 70% of which involve the loss of ODA. Many cases of PCD can be attributed to mutations in genes encoding ultrastructural components of motile cilia such as dynein subunits or proteins involved in their docking and targeting [7]–[14], central pair microtubules [15], radial spokes [6], [16] and nexin-dynein regulatory complex [17]–[21]. Less well functionally characterized are a growing group of cytoplasmic factors that are putatively involved in trafficking or stability of dynein arm components (*DNAAF1/LRRC50*, *DNAAF2/KTU*, *DNAAF3/PF22*, *DNAAF4/DYX1C1*, *HEATR2*, *LRRC6*, *SPAG1*, *ZMYND10*, *CCDC103*, *C21ORF59*) [19], [22]–[33]. Several of these Dynein Axonemal Assembly Factors, such as the case for DNAAF1-4, work directly within a heat-shock protein (HSP)-based molecular chaperone complexes to direct the proper folding of axonemal dynein subunits [29], [31].

Given the complexity of the motile cilia assembly and function, it is possible that there is an underlying conserved transcriptional programme that could be used to identify further PCD candidate genes? Expression of the core ciliogenic programme, including components of the IFT and BBSome machinery, is regulated at the transcriptional level in part by the regulatory factor X (RFX) family of transcription factors [2]. RFX proteins are essential for ciliogenesis in *C. elegans* and *D. melanogaster* [34], [35]. Of the eight paralogues that exist in mammals, *Rfx2* and *Rfx3* have been implicated in vertebrate motile ciliogenesis [36]–[39]. These proteins contain a highly conserved DNA-binding domain, which directly interacts with a consensus sequence, the X-box motif [34], [37], [40]–[42]. Identification of X-boxes in promoters of genes transcriptionally activated during ciliogenesis has been previously used for identification of putative ciliopathy candidates [43], [44].

It has been proposed that the diversity of cilia function could arise from elaboration of a core ciliary transcriptome through additional transcriptional controls [2], [45]. FOXJ1 (HFH4), a forkhead/winged-helix transcription factor, has been shown to activate gene expression required for motile cilia formation [46]–[50]. FOXJ1 is highly expressed in tissues with motile cilia [51], [52] and only motile cilia are affected in *Foxj1* null mice [46], [47]. Overexpression of *FoxJ1* is sufficient to confer some motile functions on primary cilia in both *D. renio* and *X. tropicalis* [49], [50]. Functional diversification of specific subsets of motile cilia involves other transcription factors including the homeobox NOTO for nodal cilia [53], [54], as well as MYB and nuclear MULTICILIN for multiciliated epithelia [55], [56].

Unlike their wide distribution and diverse types in vertebrates, cilia in *D. melanogaster* are very restricted. The only somatic cells with cilia in flies are sensory neurons, which have specialized ciliary dendrites for sensory reception. Only a subset of these, the proprioceptive and auditory chordotonal (Ch) neurons, possess cilia that are motile. As a result, genes encoding axonemal dynein subunits and other motility components are uniquely expressed in Ch neurons and spermatocytes. In the *Drosophila* antenna, Ch neuron ciliary motility is proposed to be part of a mechanical amplification process involved in transducing sound vibrations through the interplay of motors and transduction channels [57], [58]. This simplicity of cilia diversity is recapitulated at the transcriptional level. A single *Drosophila* Rfx member controls expression of core ciliogenic targets in all ciliated sensory neurons [59]. Although the existence of a *Drosophila* FOXJ1 orthologue had been questioned, we recently demonstrated that the diverged Fox gene *fd3F* is required for Ch neuron function [60] and fulfills a role equivalent to *Foxj1* genes in vertebrates [58]. Fd3F protein cooperates with Rfx to control expression of Ch-specific genes, including those encoding many structural components of the motility machinery such as ODA subunits (dynein heavy chain *Dhc93AB*, homolog of DNAH9/11), and IDA subunits (*Dhc16F*, homolog of *DNAH6*, and *CG6971*, homolog of *DNALI1*). In addition, Fd3F was found to regulate several unknown or poorly characterized genes that shared a similar Ch neuron-specific transcriptional profile. Some of these have subsequently been found to encode cytoplasmic proteins that are implicated in the assembly and/or transport of the dynein arm apparatus (*tilB*/*LRRC6*, *dtr*/*DNAAF1*), and their mutation in humans leads to PCD. In the majority of these target genes, conserved Fox and Rfx consensus binding motifs could be found in their promoters in close proximity to the transcriptional start site [58]. We hypothesized that this transcriptional fingerprint could be used to predict novel components of the motile cilia machinery as a route to finding additional human orthologues potentially involved in PCD.

Here, we present our studies on one such candidate, *CG31320*/*HEATR2*, which plays a conserved role in the assembly and/or stability of ODA and IDA in humans and in flies. We identify a novel splice-acceptor *HEATR2* mutation in a PCD family associated with respiratory and laterality defects. *CG31320* mutant flies exhibit aberrant proprioception, deafness and immotile sperm due to ciliary/flagella motility defects, which correlates with a lack of ODA and IDA in the cilia. Similar to the RFX- and FdF3- regulated expression of *CG31320* in Ch neurons, we show higher levels of cytoplasmic HEATR2 early in mammalian motile ciliogenesis within differentiating cells that also express high RFX3 and FOXJ1. We present human HEATR2 immunoprecipitation data showing interaction with outer arm dynein intermediate chain DNAI2, but not other DNAAFs or HSPs. We therefore propose that HEATR2 is unique amongst known DNAAFs as it acts in the early stages of cytoplasmic dynein preassembly but not in a classic chaperone/client function. We suggest HEATR2 functions as a flexible scaffold for stabilizing interactions between dynein subunits, like DNAI2, during cytoplasmic pre-assembly.

## Results/Discussion

### A novel splice acceptor mutation in *HEATR2* results in PCD

**Figure 1:**
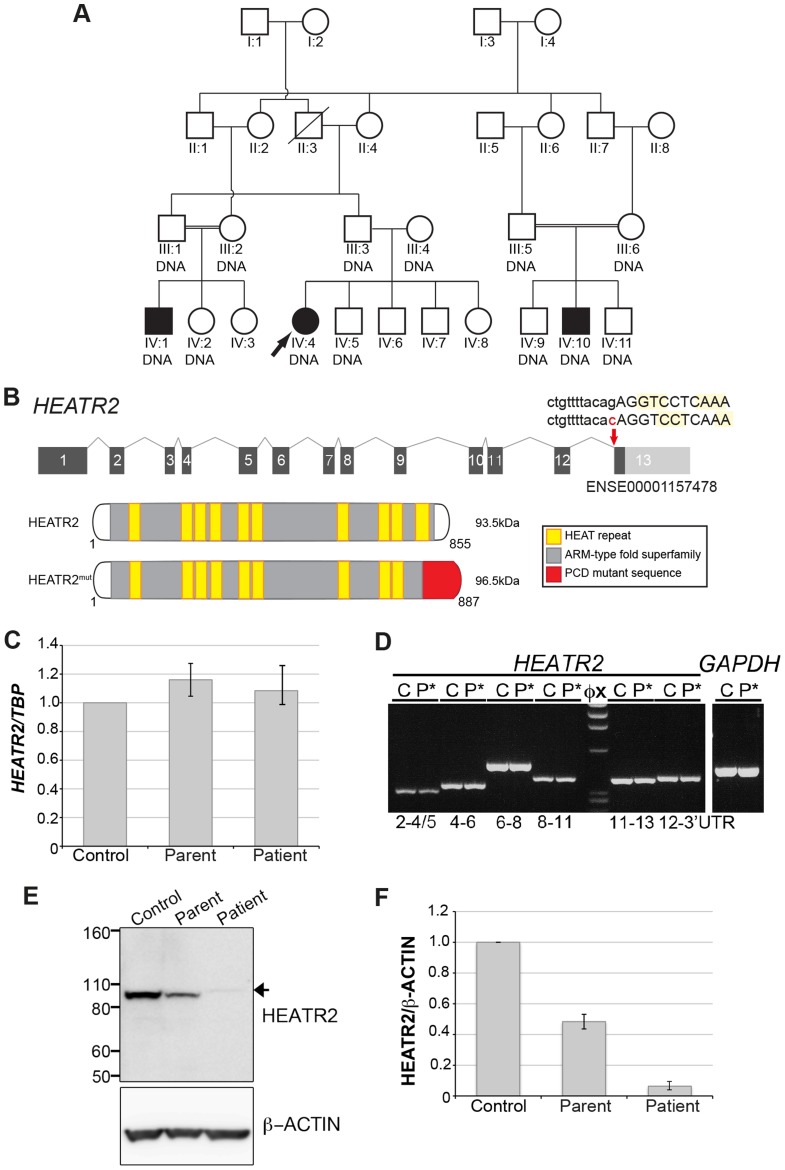
*HEATR2* splice mutation results in alteration of the final conserved HEAT repeat and protein instability. (A) Pedigree of related families of UK-Pakistani descent. IV∶4 identifies the proband, also designated by the arrow. Solid symbols (individuals IV∶1, IV∶4 and IV∶10) indicate those affected with PCD. Double lines indicate consanguineous marriages. The individuals labeled DNA signified those that had their DNA included in SNP genotyping. (B) Schematic of *HEATR2* transcript showing the transversion mutation (*ENST00000297440:c.2432-1G>C*) affecting the splice acceptor site of the final exon. The mutation results in inactivation of this splice site and utilization of an adjacent downstream cryptic splice acceptor site in exon 13, causing a 2-nucleotide AG deletion in the *HEATR2* transcript, resulting in a frameshift in translation (Figure S2B). This is predicted to alter the final 44 amino acids of the protein and add an additional 33 amino acids with creation of a novel termination signal at codon 888 in the 3′UTR (See Figure S3A). This mutation disrupts the final highly conserved HEAT repeat and alters the C-terminus of the ARM-type fold superfamily domain (red). (C) Relative expression levels of *HEATR2* transcript by RT-qPCR, when normalized to the reference *TBP* gene. (D) The PCD transversion mutation (*ENST00000297440:c.2432-1G>C*) does not affect *HEATR2* transcript stability or gross splicing as shown by RT-PCR on parental control (C) and patient (P*) cDNA from LCLs. PCR products spanning the gene including the splice acceptor mutation at Exon 11–13 and Exon 12-3′UTR show no obvious alterations in size. Direct sequencing confirmed a 2 base pair deletion consistent with efficient splicing to the cryptic splice acceptor at the start of exon 13 in PCD patients (Figure S2B). (E) Western blot analysis on total protein extracts from unrelated control, heterozygous parental and homozygous patient LCLs demonstrates the PCD mutation (*ENST00000297440:c.2432-1G>C*) results in an elongated HEATR2 protein present at reduced levels implying instability. The slight shift in mobility of the protein in the patient is consistent with the predicted 3 kDa size shift due to the amino acid alterations described. β-actin is used as a loading control. (For longer exposure see Figure S3B). (F) Levels of HEATR2 protein normalized relative to β-actin reveal that parental samples which are heterozygous for the mutation shows a reduction to ≈50% of that of unrelated controls whilst the homozygous patient sample shows a reduction to ≈3% of control levels.

We identified an extended UK-Pakistani family with three affected children presenting with PCD (Figure 1A). The proband (IV∶4) presented at the age of 3 with chronic respiratory infections, middle ear disease and chronic nasal discharge (Table 1). She had also experienced neonatal respiratory distress and dextrocardia was apparent on chest X-ray. Nasal ciliary biopsy confirmed the PCD diagnosis by video microscopy, and written reports of transmission electron microscopy revealed absence of both IDA and ODA (data not shown). Her cousins subsequently presented at the age of 2 (IV∶1) and 4 (IV∶10) with chronic chest infections and nasal discharge (Table 1). The diagnosis of PCD was confirmed in both these latter cases by nasal ciliary biopsy, again electron microscopy confirmed absence of both inner and outer dynein arms.

**Table 1:** Clinical characteristics of the UK-Pakistani PCD-affected subjects with HEATR2 mutations.

Subject	Gender	Age	Clinical Manifestations				Ultrastructural Defect
			Lung:	Upper airway:	Middle Ear:	Laterality:	
IV∶4	F	3	NRD, CB, CC, BS	RS	OM	DC	Absent IDA & ODA
IV∶1	M	2	NRD, CB, CC	RS	N	SS	Absent IDA & ODA
IV∶10	M	4	CB, CC	RS	OM	SS	Absent IDA & ODA

**Key:**

The following abbreviations are used: Age, age at presentation; M, male; F, female; NRD, neonatal respiratory distress; CB, chronic bronchitis/pneumonia; CC, chronic cough; BS, established bronchiectasis; RS, chronic rhinosinusitis; OM, chronic otitis media; N, no; DC, dextrocardia; SS, *situs solitus*; IDA, inner dynein arms; ODA, outer dynein arms.

Whole genome SNP autozygosity mapping in this consanguineous family identified a single concordant homozygous region of 2.6 Mb on chromosome 7∶46,239–3,179,991 (GRCh37) (Figure S1A) shared by all three affecteds and not seen in unaffected sibs. A custom oligonucleotide array was designed to capture all exons as well as intron/exon boundaries within the interval, and these were analyzed by Next Generation Sequencing. This identified a single potentially pathogenic variant c.2432-1G>C in the splice acceptor site of the final exon of *HEATR2* (NM_017802). Sanger sequencing confirmed the segregation of the variant with the phenotype within the family (Figure S1B). The change which affects the highly conserved AG of the consensus U2-type splice acceptor (Figure S2A) is absent in 176 ethnically matched control individuals as well in 6503 European and African American subjects in the Exome Variant Server (NHLBI GO Exome Sequencing Project (ESP), Seattle, WA (http://evs.gs.washington.edu/EVS/) (November, 2013 accessed)). With a total of 13,358 genotyped control chromosomes, we could significantly achieve greater than 95% power to exclude the likelihood that this is an uncommon SNP with an allele frequency of 0.1% [61], strongly indicating that this is the PCD causative mutation. All *HEATR2* exons were sequenced in an additional 23 PCD patients but no further mutations were detected. However, another PCD-causing *HEATR2* allele has been recently reported among a Midwest American Amish pedigree which contains a missense Leu795Pro mutation in a highly conserved residue in exon 12 [26]. Together, these studies indicate that independent mutations in *HEATR2* contribute to a small proportion of PCD cases.

We sequenced *HEATR2* cDNA from patients to investigate the consequences of the G-to-C transversion mutation on *HEATR2* transcripts. The PCD transversion mutation (*ENST00000297440:c.2432-1G>C*) affects the terminal G of the AG at the end of intron 12/13 located within the exon 13 splice acceptor consensus sequence. In patients, the first two coding bases of exon 13, also AG, create a cryptic splice site immediately adjacent which is utilised, resulting in a two base pair deletion of *HEATR2* transcript and a consequent coding frameshift within exon 13 (Figure S2B). The mutation does not affect mutant *HEATR2* transcript stability as shown by RT-qPCR and RT-PCR from parental control and patient cDNA (Figure 1C, D), with the heterozygous parental samples containing approximately equal levels of each splicing variant (Figure S2B). RT-PCR products spanning the splice acceptor mutation, exon 11–13 and exon 12-3′UTR showed no significant alterations in size, consistent with efficient splicing to the cryptic splice acceptor at the start of exon 13 in PCD patients and loss of just 2 bp (confirmed by sequencing) (Figure 1D, S2B). Moreover, ribonuclease protection assay (RPA) using riboprobes containing portions of exons 12 and 13 from unaffected control and patient cDNA confirmed with high sensitivity and specificity these splicing events are occurring in mutant *HEATR2* transcripts (Figure S2C).

This PCD mutation and consequent frameshift (*c.2432-2433delAG*) was predicted to replace the final 44 amino acids of HEATR2 protein with 77 novel amino acids (*pGlu811GlyfsTer78*: Figure S3A). This mutation would disrupt the last of ten highly conserved HEAT repeats and alter the C-terminus of the ARM-type fold superfamily domain (Figure 1B). This simple array of repeating motifs is found in α-solenoid proteins and is best characterized by β-importin with 19 HEAT repeats. They are believed to create a highly flexible macromolecule with large surface area key for mediating protein-protein binding, both in terms of cargo selection and interactions with cell transport machinery [62]. Western blot analysis confirmed the PCD HEATR2 mutation (*pGlu811GlyfsTer78*) resulted in a slight shift in mobility consistent with the predicted 3 kDa size increase. More striking, however, was a striking reduction in total HEATR2 levels in the patients (Figure 1E, S3B). Parental samples, heterozygous for the mutation, showed a ≈50% reduction in β actin-normalized HEATR2 levels compared to unrelated controls whilst the homozygous patient samples were ≈3% of control levels (Figure 1F). This suggests the mutation (*pGlu811GlyfsTer78*) resulted in pathogenic changes in the amino acid sequence and C-terminal structure of HEATR2 protein resulting in its instability. Our study supports the recent report [26] of *HEATR2's* contribution to the genetic heterogeneity underlying Primary Ciliary Dyskinesia.

#### *CG31320* is expressed in a FOX- and RFX-dependent manner

**Figure 2:**
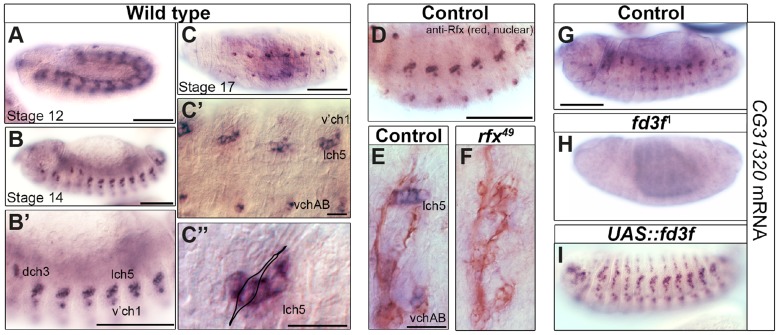
The *HEATR2* orthologue *CG31320* is highly expressed in motile ciliated mechanosensory Ch neurons in an Rfx- and Fox-dependent manner. (A–D) *In situ hybridization* shows that *CG31320* mRNA is present in chordotonal (Ch) neurons from about stage 12, when transient mesoderm expression is also observed (A). Through early neuronal differentiation (stage 14: B, B′ (higher magnification) to late neuronal differentiation (stage 16: C, C′), *CG31320* expression is highly expressed in a restricted pattern to Ch neurons. (C″: higher magnification of C, stage 16). Overlay of a Ch neuron schematic illustrating strong and restricted *CG31320* expression in these clusters of ciliated mechanosensory neurons in late stage embryos. (Scale bars: B, B′,C: 100 µm; C′,C″: 20 µm) (D) Double labeling of stage 16 wild type embryos for anti-Rfx (red, nuclear stain) and *CG31320* mRNA (diffuse blue) shows co-labelling between Rfx and *CG31320* in late Ch neurons. The master ciliogenic transcription factor Rfx is expressed transiently in all sensory neurons but at this stage it is only present in the motile ciliated Ch neurons. (Scale bar: 100 µm). (E,F) *CG31320* expression is dependent on the *Rfx* transcription factor. *CG31320* mRNA expression (blue) is lost in Ch neurons (anti-HRP: neuronal glycoproteins, red) of *rfx^49^* mutants [35]. (Scale bar: 20 µm). (G–I) Fd3F is necessary and sufficient to drive *CG31320* expression in sensory neurons. Compared to control (G), *CG31320* expression is abolished in homozygous *fd3F^1^* mutant embryo (H). (I) Conversely, ectopic *fd3F* expression (*scaGal4*, UAS-*fd3F*) expands *CG31320* expression into other ciliated sensory neurons in stage 16 embryos. (Scale bar: 100 µm).

Our interest in transcriptional targets of the ciliary motility programme [58], [60] independently led us to the *Drosophila HEATR2* orthologue *CG31320*. High-resolution temporal gene expression profiling during *Drosophila* neural development suggested that *CG31320* is expressed in differentiating Ch neurons prior to cilium formation [60]. We proceeded to confirm the embryonic expression pattern of *CG31320* by RNA *in-situ* hybridization. *CG31320* mRNA was restricted to Ch neurons with expression beginning from about stage 12, after neuronal specification but preceding cilium formation (Figure 2A), and continuing throughout neuronal differentiation (stage 14: Figure 2B–C″). Co-staining with anti-Rfx confirmed co-expression in late stage developing Ch neurons (Figure 2D).

Given its expression in early differentiating Ch neurons, we next investigated whether *CG31320* is regulated by ciliary transcription factors. We found that *CG31320* expression is Rfx-dependent, since *CG31320* expression is lost in *Rfx* mutant Ch neurons (Figure 2E,F). *CG31320* expression in Ch neurons was also *fd3F*-dependent, as it was absent in *fd3F* mutants (Figure 2G,H; [58]) and could be induced ectopically by forced Fd3F expression in all the Rfx-positive non-motile ciliated sensory neurons (Figure 2I). These studies demonstrate that *CG31320* expression is restricted in a *Rfx-* and *fd3F*-dependent manner to the neural cells that form specialized motile cilia. Outside the Ch lineage, *CG31320* expression was also detected transiently in early stage 12 non-ciliated mesoderm (Figure 2A) and in the adult testes (http://flybase.org/reports/FBgn0051320.html); its transcriptional control in these lineages remains unclear.

**Figure 3:**
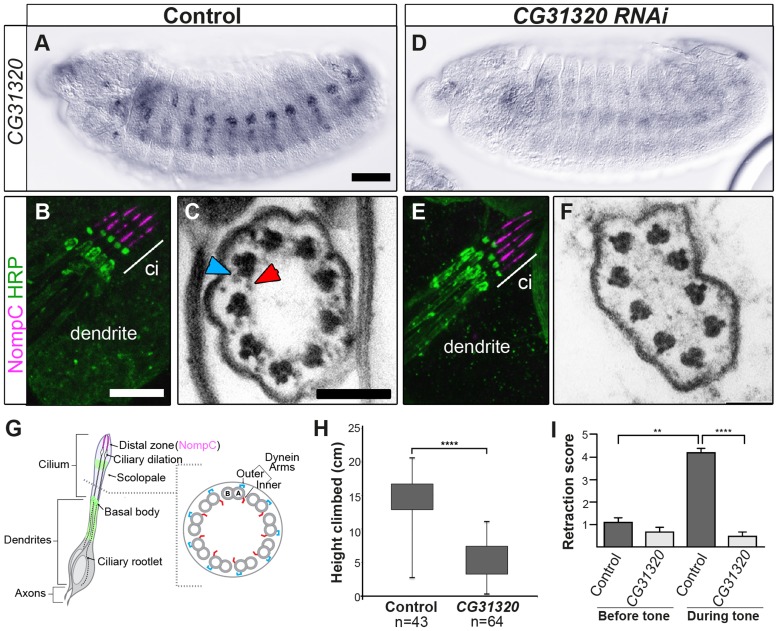
*CG31320* is required for mechanosensory structure and function in Ch neurons. (A–C) Control and (D–F) Sensory-neural specific *CG31320* RNAi knock-down (*UAS-Dcr2; scaGal4/UAS-CG31320 RNAi*). (A,D) *In-situ* hybridisation confirms that substantial loss of *CG31320* mRNA was achieved by the knock-down. (Scale bar: 50 µm). (B,E) Immunofluorescence of larval Ch neurons using the pan-neuronal marker (anti-HRP: green, neuronal glycoproteins marking luminal bands at level of basal body and close to ciliary dilation) and the ion channel NompC/TRPN1, (anti-NompC; magenta; marks the distal non-motile cilium tip) shows that loss of *CG31320* results in no gross cilia dysmorphology or loss of compartmentalization of ciliated Ch structures. (Scale bar:10 µm). (C,F) TEM of Ch cilia cross-sections from adult antennae (Johnston's organ), showing nine axonemal microtubule doublets shown schematically in (G). (C) The electron-dense structures corresponding to inner (red arrowhead) and outer (blue arrowhead) axonemal dynein arms are clearly seen in wild-type. (F) These are not observed in *CG31320* knock-down cilia. (Scale bar: 100 nm). (G) Schematic illustration of Drosophila Ch neurons showing the localisation of markers in the cilia and the presence of dynein arms in the proximal motile zone. (H) Ch neuronal function is measured by the negative geotaxis climbing assay for adult flies. The height climbed by control (n = 43) versus *CG31320* RNAi flies (n = 64) reveals the latter to be uncoordinated. (Mann-Whitney U test: *P*≤0.0001). (I) *CG31320* RNAi knock-down larvae do not respond in an auditory assay. Retraction score is the number of larvae (in a sample of 5) exhibiting head shortening during a 0.5 second time window. Shown is the mean retraction score for several tests (control: n = 15; RNAi: n = 16); error bars are standard error of the mean. Statistical analysis was performed using the Friedman test followed by Dunn's multiple comparison post-hoc test. (** represents *P*≤0.01; **** represents *P*≤0.0001).

To determine whether *CG31320* is required for ciliary motility, we generated *CG31320* mutant flies by imprecise excision of an associated P element in the line *CG31320^EY06677^*. The resultant excision line *CG31320^27^* had a 992-bp deletion of the 5′ end of the gene, including the transcriptional and translational start sites, and *CG31320* mRNA was absent in homozygous *CG31320^27^* embryos (Figure S4). Visual inspection of deletion mutants showed a complete lack of surviving homozygote adult flies while homozygote larvae were smaller but with no obvious morphological defects. Since Ch neuron dysfunction is not lethal, this suggests a vital non-cilial role for *CG31320* correlating with its expression in the developing midgut, which may affect nutrition. To bypass this lethality and focus on the cilial role, we used a Gal4-inducible RNAi line (P{KK102625}VIE-260B) to generate knock-downs lacking *CG31320* specifically in embryonic and adult developing sensory neurons (*scaGal4 UAS-Dcr2/UAS-CG31320-KK102625 RNAi*). RNAi generated knock-down embryos showed a strong reduction in neural *CG31320* mRNA (Figure 3A,D). These knock-down flies are viable but uncoordinated, performing poorly in climbing assays (Figure 3H), suggesting that Ch neurons are defective as these are required for proprioception during coordinated locomotion. Ch neurons are also required for hearing, and so we tested for larval auditory function. Control larvae contract abruptly when exposed to a 1-kHz tone, due to auditory reception by Ch neurons (Figure 3I). In contrast, *CG31320* knock-down larvae failed to respond to the tone.

Despite these functional defects, the specification and gross differentiation of Ch neurons in *CG31320* RNAi flies was unaffected (Figure 3). Moreover, loss of *CG31320* does not grossly disrupt formation of Ch neuron cilia or their functional compartmentalization, as shown by immunofluorescence for a neuronal marker (anti-HRP: green) and the ion channel NompC/TRPN1, which marks the distal non-motile cilium tip (anti-NompC; magenta) (Figure 3B,E,G). We examined Ch neuron ultrastructure in the antenna of *CG31320* knock-down adult flies by transmission electron microscopy (TEM). This revealed that the normal 9+0 arrangement of microtubule doublets in the proximal (motile) zone of the Ch neuron cilium was present but the axoneme lacked both ODA (blue arrowheads) and IDA (red arrowheads) (Figure 3C,F,G; Table 2). Structures consistent with remnant dynein arms were only ever detected on a small minority of doublets. Thus, *CG31320* is required specifically for the presence of the Ch ciliary motility apparatus.

**Table 2:** Frequency of visible dynein arms in *Drosophila* sensory-neural specific *CG31320* RNAi mutant Ch neuronal axonemes by TEM.

	*scaGal4*/+ (control)	*scaGal4*/UAS-*CG31320^KK102625^*	*P* (Fisher's)
	n = 63	n = 126	
Outer dynein arm	97%	6.30%	****P≤0.0001
Inner dynein arm	91.50%	3.20%	****P≤0.0001

**Key:**

n: number of axonemal microtubule doublets scored. Visible dynein arm: the percentage (number) of axonemal microtubule doublets for which staining consistent with a dynein arm is visible. In both instances, Fisher's Exact Test shows significant reduction in the knock-down cilia (*P*≤0.0001).

**Figure 4:**
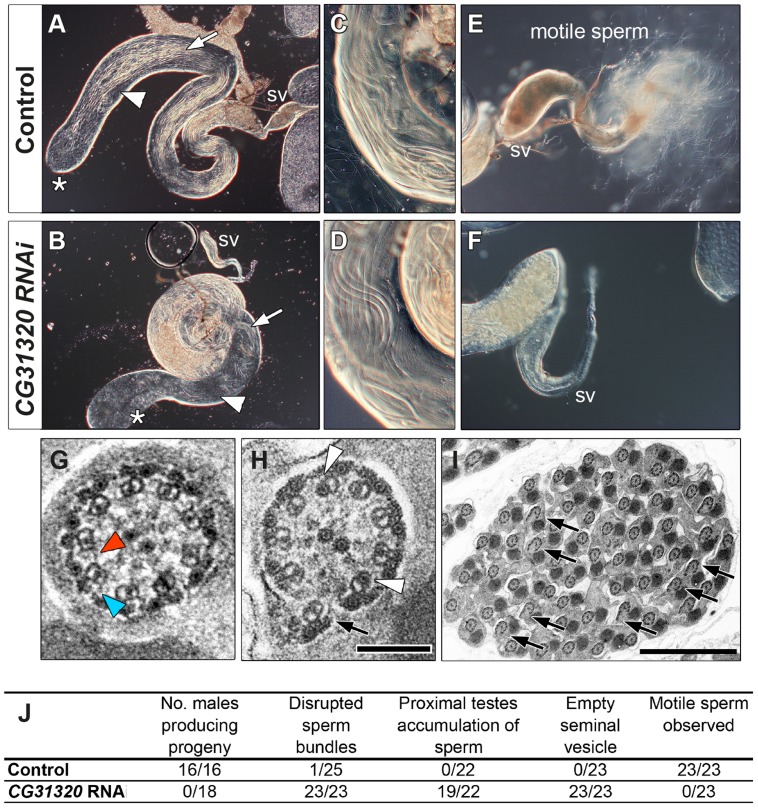
*CG31320* is required for sperm flagellar motility and male fertility. (A,C,E) Control and (B,D,F) *CG31320* testes-specific inducible RNAi knock-down (*UAS-Dcr2; UAS-CG31320 RNAi; Bam-VP16-Gal4*) adult flies. (A,B) Upon knock-down, gross morphology of the testis and seminal vesicle (SV) is normal and sperm bundles can be observed in control and knock-down testes (arrows). (C,D) Higher magnification views show normal organization of developing sperm bundles in the proximal testis in *CG31320* knock-down. (E,F) In control (E), the seminal vesicle is full of mature sperm and many motile spermatozoa are visible swimming away, (F) Mature sperm are not visible within *CG31320* knock-down seminal vesicles and no motile sperm are observed upon its dissection. (G–I) TEM images of adult testes post-elongation flagellar transverse sections show (G) a control spermatid, with dynein arms visible on some of the microtubule doublets (colored arrowheads) whilst (H) *CG31320* RNAi mutants lack dynein arms. Despite a normal “9+2” configuration, some mutant “A” doublet sub-tubules have electron-dense cores (white arrowheads) or disruptions suggesting defects in nexin links between AB doublets (arrows). (I) Transverse section of *CG31320* RNAi knock-down mutant spermatid cyst highlights frequency of axonemal disruption (arrows). Scale bars: 100 nm (G,H), 2 µm (I). (J) Summary of male fertility phenotypes between control and *CG31320* testes-specific RNAi knock-down. Number of males producing progeny represents progeny from crosses to wild-type females. The knock-down flies were completely infertile, even though mating was observed. In addition to empty seminal vesicles, knock-down testes exhibit accumulation of sperm and some disruption of sperm bundles. These phenotypes appear to represent secondary consequences of the failure of sperm to move to the seminal vesicle, which requires sperm motility.

*CG31320* is also expressed in the adult testes, which contains the only other motile cilium-like structure in *Drosophila*, the sperm flagellum. Transcriptional control of ciliary motility in testes is unclear. Although Rfx is expressed in spermatids, *Rfx* mutant spermatozoa are motile but males are too uncoordinated to mate. Similarly, *fd3F* mutant males are fertile [58]. Moreover, extension and maintenance of the *Drosophila* sperm flagella is IFT-independent, such that IFT-B mutant sperm are motile and structurally normal. Thus the assembly of a 9+2 sperm axoneme with ODA and IDA occurs by an alternate cytosolic assembly mechanism [63], [64]. To address whether *CG31320* function is also required for sperm motility, we generated testes-specific *CG31320* RNAi mutant males (*Bam-VP16-Gal4; UAS-Dcr2/UAS-CG31320-KK102625 RNAi*). Although such males can mate, they are sterile (n = 20 males tested) (Figure 4J). We examined sperm development in control and mutant testes. Normally spermatogonial germ cells at the apical tip of testes (Figure 4A asterisk) go through 4 synchronous mitotic amplifications with incomplete cytokinesis to produce a cyst of 16 interconnected spermatogonia (or primary spermatocytes). Eventually all 16 spermatocytes undergo meiosis I and II, to form a cyst of 64 inter-connected primary spermatids (Figure 4A arrowhead). Formation of sperm axonemes yields a bundle of extremely long spermatids (Figure 4A (arrows) and 4C) that stretch almost the entire length of the testis. The final step in spermatogenesis is a highly complex process of membrane remodelling called individualisation to yield 64 individual sperm that are then transferred to the seminal vesicle (SV) in a process which appears to be dependent on sperm motility. *CG31320* knock-down testes appeared to develop normally and mature sperm were clearly present (Figure 4B, D), but no motile sperm were observed in the SV (Figure 4F: SV with motile sperm in 0/23 mutant testes examined compared with 23/23 control testes).

To investigate the cellular defect underlying *CG31320* mutant sperm immotility, we carried out TEM analysis of spermatid cysts. While wild type cysts contain 64 spermatids produced by division of a single precursor, *CG31320* knock-down cysts contained an average of 60.7 identifiable spermatids (s.d. = 3.39, n = 6). These had a normal axonemal “9+2” arrangement, but dynein arms were generally not visible (colored arrowheads: Figure 4G, H), consistent with the lack of sperm motility. In addition, a proportion of axonemes showed separation of some doublets from the core (arrows, Figure 4H, I), suggesting defects in motility-associated nexin links. In addition some “A” sub-tubules of the doublets contained electron-dense cores, which are normally only seen in the accessory and central pair microtubules (open arrowheads, Figure 4H, I). This constellation of ultrastructural defects has been previously reported for *tilB* (*LRRC6*) and *CG11253* (*ZMYND10*) mutant sperm [23], [65]. Despite the fundamental differences between how Ch neuron and sperm flagella axonemes are built, similar ODA and IDA defects in both demonstrate *CG31320* plays a core role in assembly of the ciliary motility apparatus.

### *CG31320/HEATR2* is a highly conserved gene with a ciliary motility signature

**Figure 5:**
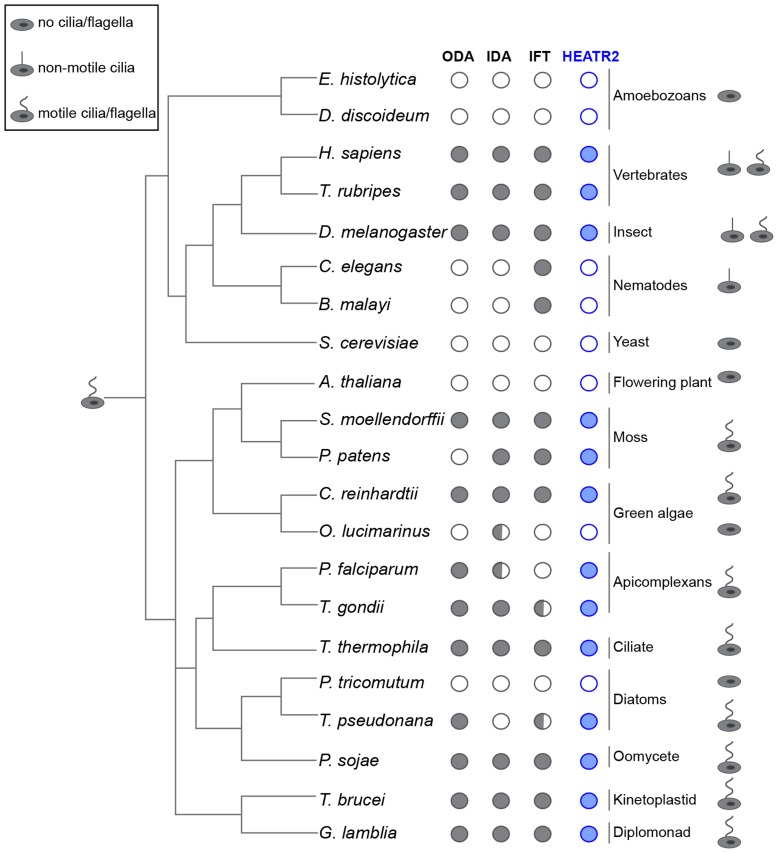
*CG31320/HEATR2* is conserved in eukaryotes with motile cilia/flagella and its associated axonemal dynein apparatus. *CG31320/HEATR2* orthologues are found in species with cilia/flagella that have motile function and retain elements of the axonemal dyneins required for this motility. Species which have no cilia (*ie.* amoebozoans, flowering plants, yeast) or those which lack motile cilia (*i.e.* nematodes) have lost *HEATR2* orthologues as well as the axonemal dynein genes. Interestingly, unusual species with variant motility programmes still retain *HEATR2* orthologues. These include *T. pseudonana* whose male gametes have motile axonemes without inner arm dyneins, and *P. patens*, whose male gametes have motile flagella without outer arm dyneins. Similarly, *P. falciparum* which assembles its flagella intracytosolically through an IFT-independent programme, retains a *HEATR2* orthologue. This suggests *CG31320/HEATR2* is an essential element of an ancient programme required for ciliary/flagellar motility. This figure is a summary of a more extensive search detailed in Table S1 for *CG31320/HEATR2* orthologues as well as axonemal dynein components of the outer (ODA) and inner (IDA) dynein arms, as summarized in columns. Filled circles: orthologues as determined by the top score in reciprocal BLASTP or TBLASTN searches. Open circle: no homologue present. Half-filled circle: evidence supporting existence of at least one orthologue per category as analyzed in Table S1 by reciprocal BLASTP or TBLASTN searches. Information for the intraflagellar transport (IFT) pathway built upon Wickstead and Gull (2007) with our own searches for IFT components by reciprocal BLASTP or TBLASTN searches.

Our studies indicated that *CG31320* in *Drosophila* is associated with ciliary motility function and identification of human *HEATR2* PCD disease mutations suggested this function is conserved. We examined whether the co-distribution of *CG31320* orthologues with representative components of axonemal dynein across the eukaryotic evolutionary tree supported a specific role in the assembly or stability of structures required for motile cilia function (Figure 5, Table S1). We found *CG31320/HEATR2* orthologues, as well as components of both ODA and IDA, to be absent from all non-ciliated lineages such as yeast but importantly also absent from lineages possessing only non-motile cilia such as nematodes [66]. *CG31320/HEATR2* orthologues and components of both IDA and ODA were identified in all lineages that have motile cilia or flagella at some point in their life cycle, with a few conspicuous exceptions. A *CG31320/HEATR2* orthologue was found in the marine centric diatom *Thalassiosira pseudonana*, which has motile sperm whose 9+0 axonemes bear only ODAs [66]. Conversely, a *CG31320/HEATR2* orthologue was also identified in the moss species *Physcomitrella patens*, which has motile sperm with axonemes bearing only IDAs. This supports the above finding that *CG31320/HEATR2* is functionally required for the correct assembly of both types of dynein arms. Surprisingly, we identified a conserved *CG31320/HEATR2* orthologue in the non-motile green algae *Chlorella variabilis*, which is assumed to be asexual. However, the existence of several meiosis-specific and flagellar genes in this organism including subunits of outer arm dyneins, has led to the suggestion that some flagellar-derived structure involved in sexual reproduction may have been retained [67]. This evolutionary pattern of co-conservation of *CG31320/HEATR2* with the ciliary motility machinery, together with the loss of IDA and ODA observed in *CG31320* mutant axonemes, suggests *CG31320* is an ancient component of the ciliary/flagellar motility programme required for both IDA and ODA.

**Figure 6:**
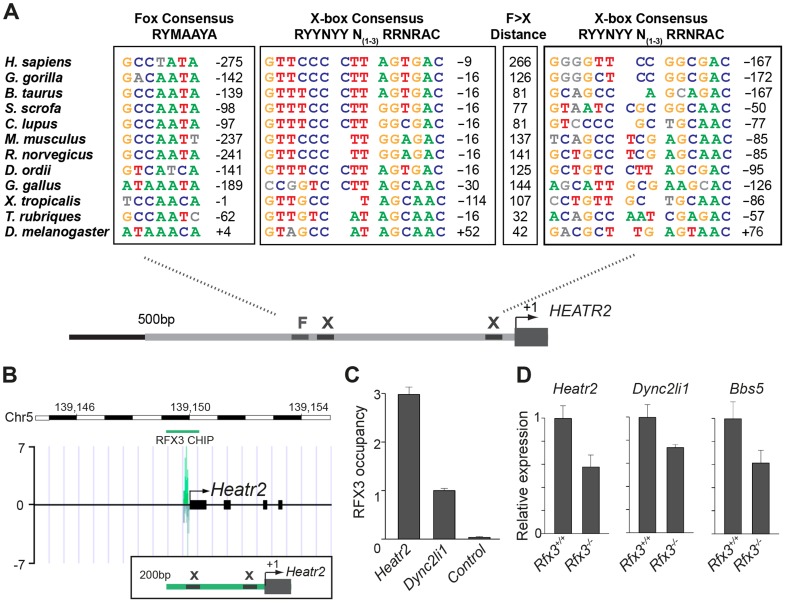
*CG31320*/*HEATR2* orthologues share conserved upstream regulatory FOX motifs and X-boxes of a master cilia motility transcriptional programme. (A) Using the human upstream epigenetic markings and conservation to mouse and rat to define conserved predicted regulatory elements, we focused analysis on the 500 bp upstream of the *HEATR2* ATG and syntenic regions in other species to identify X-box sequences, along with the nearest conserved FOX motifs. These sequences are coloured where they conform to recognized core consensus sequences for generic FOX proteins (RYMAAYA [71]) and RFX (RYYRYYN_(1–3)_RRNRAC [42]). Nucleotides are shown in grey if they vary from the consensus. Note for the second identified X-box site the 3′ site is extremely well-matched whilst the 5′ half-site is often more degenerate [37], [58]. The distance from the Fox motif and X-box to the transcription start site is indicated, or else the distance to the ATG is indicated if a sizeable 5′UTR is present (*i.e. D. melanogaster, C. lupus*). An expanded table of the analysis is provided in Table S2. (B) ChIP-Seq data reveals a single, specific RFX3 peak 200 bp upstream from the transcriptional start site in OF1 mouse primary differentiated ependymal cell culture. Insert illustrates the two X-boxes bioinformatically predicted within the peak sequence. (C) Directed ChIP-qPCR data validates RFX3 occupancy is enriched at *Heatr2* promoter in OF1 cells, normalized to known target gene *Dyn2li1* and relative to a control sequence, downstream region in the *Dync2li1* gene. (D) *Heatr2* expression is ≈55% reduced in *Rfx3^−/−^* ependymal cells similar to reductions in expression observed for two known direct Rfx3 targets, *Dync2li1* and *Bbs5*. qPCR data represent the average of three different assays performed in triplicate ± SEM. All data are considered significant using Student's t-test. (*Heatr2 P* = 0.003652632; *Dync2li P* = 0.013123897; *Bbs5 P* = 0.022511438).

We further investigated whether FOX/RFX ciliary motility transcriptional signature used to identify *CG31320* was also conserved amongst orthologues. Analysis of promoter sequences of *CG31320/HEATR2* orthologues in several vertebrate genomes revealed the presence of highly conserved X-boxes situated within 500 bp upstream of the transcriptional start site (Figure 6A, Table S2). In all species examined, very close to the start of *Heatr2* transcription, we identified most often in the same position (−16), one palindromic RFX (X-box) binding site (RYYNYY N_(1–3)_ RRNRAC: [42]) extremely well-matched in both the 5′ and 3′ half sites. Interestingly, a second sometimes more degenerate X box was also identified in close proximity (15–162 bp away, relative position more variable between species Table S2), where the 5′ half-site was more degenerate. This second “relaxed” motif has previously been reported in several RFX targets that are components of the motile machinery in both flies and mammals [37], [58] and suggests the existence of multiple alternative DNA-recognition modes among members of the RFX family of proteins [68]. Although the significance of multiple X-box motifs in regulating target gene expression is unclear, it has been proposed that they may “fine-tune” the level and spatial expression of targets [69], [70]. Initial analysis using a stringent FOXJ1 consensus site (WDTGTTTGTTTA or KTTTGTTGTTKTW: [51]) revealed no upstream sites in close proximity to the transcriptional start in all vertebrate promoters, however using a less rigorous consensus core motif used by the majority of forkhead proteins (RYMAAYA [71]) and used in our *Drosophila* studies [58], we were able to identify conserved motifs in this 500 bp upstream regulatory region. These were similar to motifs for FOXJ1 recently defined by protein-binding microarray [72]. These studies suggest that the co-operative transcriptional control of *CG31320/HEATR2* by FOX and RFX factors as part of the ciliary motility programme may also be widely conserved.

To examine the physiological relevance of these putative binding sites in the regulation of *Heatr2* expression, we examined an RFX3 ChIP-seq data set from differentiated mouse primary ependymal cell cultures which bear motile, multicilia [37]. ChIP-seq analysis identified a single unique RFX3-peak within 5 kb of the transcriptional start site of *Heatr2* (Figure 6B), a region that contained both the predicted X-boxes. This is similar to peaks for the known RFX3-target gene *Dync2li1*(Figure S5A), with a canonical conserved X-box in close proximity to the transcriptional start site (Figure S5B) and strongly suggests that the X-boxes are occupied during motile ciliogenesis. ChIP-qPCR validation revealed strongly enriched RFX3 occupancy at the *Heatr2* promoter comparable to or greater than validated target *Dync2li1* target sequence (Figure 6C). Consistent with these data, *Heatr2* expression in *Rfx3^−/−^* ependymal cells is decreased ∼55% compared to controls, similar to that which is observed for known targets *Dyn2li1* and *Bbs5* (Figure 6D). Together, these analyses indicate that mouse *Heatr2*, as in *Drosophila*, is directly regulated in part in an RFX-dependent manner.

**Figure 7:**
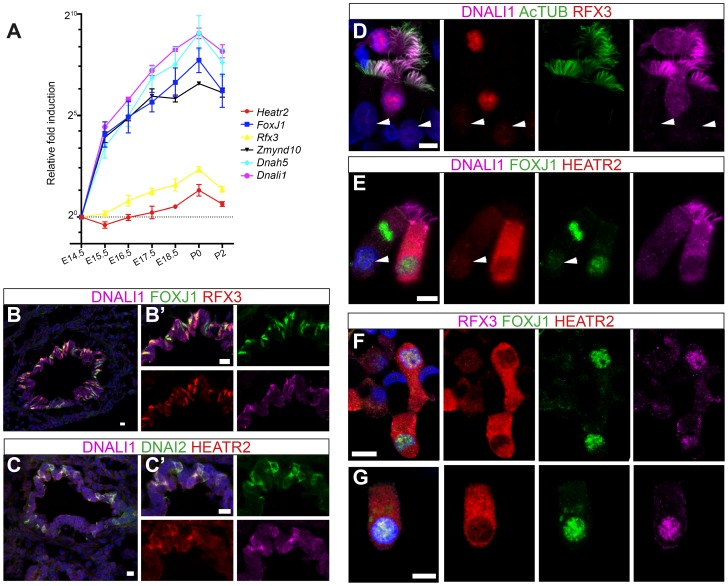
HEATR2 is highly expressed in tissues with motile cilia. (A) Developmental changes in gene expression were assayed by RT-qPCR on RNA extracted from wild-type mouse lungs and trachea from E14.5-P2 (N = 3 independent biological samples for each time-point). qPCR data represents the average of three different assays for three samples performed in triplicate ± SEM. Kruskal-Wallis non-parametric analysis of variance was performed and was significant for all genes (*Zmynd10 P* = 1.558e^−08^; *Dnahc5 P* = 1.141e^−05^; *Dnali1 P* = 9.814e^−06^; *Foxj1 P* = 1.956e^−09^; *Heatr2 P* = 1.604e^−06^; *Rfx3 P* = 6.68e^−06^). (B,C) Immunostaining on sections of E15.5 mouse lungs, where strong RFX3 and FOXJ1 signals are co-expressed in a “salt and pepper” pattern only in large proximal airways, not smaller, more distal airways (see Figure S6A,B). Although they are not yet multiciliated, these cells also express components of axonemal dyneins and high levels of HEATR2 in their cytoplasm. (Scale bar: B,C = 50 µm, B′,C′ = 10 µm). (D–G) Immunostaining of human nasal brush epithelial cells for: (D) RFX3 (HPA: red), acetylated tubulin (green) and DNALI1 (SC: purple); (E) HEATR2 (Novus: red), FOXJ1 (green) and DNALI1 (SC: purple); and (F,G) HEATR2 (Proteintech: red), FOXJ1 (green) and RFX3 (SC: purple). White arrowheads highlight fully mature motile, multiciliated cells (MMCs) that express lower nuclear RFX3 and FOXJ1 with reduced HEATR2 and with axonemal dynein components entirely in cilia. Arrows highlight immature MMCs for comparison. HEATR2 is entirely cytoplasmic at all stages examined (Scale bar: D–G = 10 µm).

To further explore developmental transcriptional control of mammalian *Heatr2* during formation of motile multiciliated cells (MMCs), we first used RT-qPCR during mouse embryonic trachea and lung development. Ciliation in developing mouse airway epithelia occurs in a distinct spatial and temporal manner, from E14.0 when the first few *FoxJ1*-positive epithelial cells emerge in a proximal-distal sequence [73]. Surface multicilia are subsequently detected from E16.5 [52], [73], [74], becoming more abundant in the airway epithelia with longer cilia as development progresses. RT-qPCR analysis of E14.5 through to P2 mouse trachea and lungs revealed *Dnah5*, *Dnali1* and *Zmynd10* to have exponential expression curves during development (similar to *FoxJ1*), whilst *Rfx3* and *Heatr2* followed more linear increases in gene expression (Figure 7A). Both *Rfx3* and *Heatr2* were detected prior to *FoxJ1* expression, but a highly significant two fold increase in *Heatr2* expression was observed during *Foxj1*-dependent differentiation (*Heatr2*: 2.73±0.501 (SEM), E14.5 vs. P0: One-way Kruskal-Wallis test P<0.001, Mann-Whitney U-Test P<0.01). We next refined the spatial expression pattern of HEATR2, RFX3 and FOXJ1 by immunofluorescence in the developing bronchial epithelium at E15.5, prior to multiciliation (Figure 7B, C compared with Figure S6A,B). During this proximal-to-distal wave of differentiation, only the larger proximal airways had interspersed cells expressing high levels of FOXJ1 and RFX3, as well as subunits of inner and outer arm dyneins DNALI1 and DNAI2. These same cells expressed high levels of HEATR2. At other stages and sites of MMC differentiation, HEATR2 is expressed in a “salt-and pepper” pattern, including E18.5 epithelial cells of trachea and bronchus (Figure S6C,D), as well as in ependymal cells lining the lateral ventricles of P5 brains and MMCs lining the adult oviduct ampulla (Figure S6E, F). For comparative analysis of human cilia, we used asynchronous nasal brush epithelial cells from healthy controls, in which we were able to distinguish both immature cells (arrow) alongside terminally differentiated and fully ciliated mature cells (arrowhead). This revealed that HEATR2 expression was highest in immature cells in the process of extending multicilia, which were also those expressing higher nuclear RFX3 and FOXJ1 and predominantly cytoplasmic DNALI1 (Figure 7D–G). In adjacent fully mature MMCs, when DNALI1 was predominantly axonemal, comparatively lower levels of HEATR2 were observed, as well as lower RFX3 and FOXJ1 expression (arrowheads Figure 7D,E). Together, these results suggest that while *Heatr2* expression in mammals has evolved more complex transcriptional control compared to flies, the conserved FOX/RFX ciliary motility signature we identified is still used to drive dynamic high level expression at a developmental window when axonemal dynein pre-assembly is occurring in the cytoplasm.

### *CG31320/HEATR2* is a cytoplasmic factor required for dynein arm assembly and/or stability

**Figure 8:**
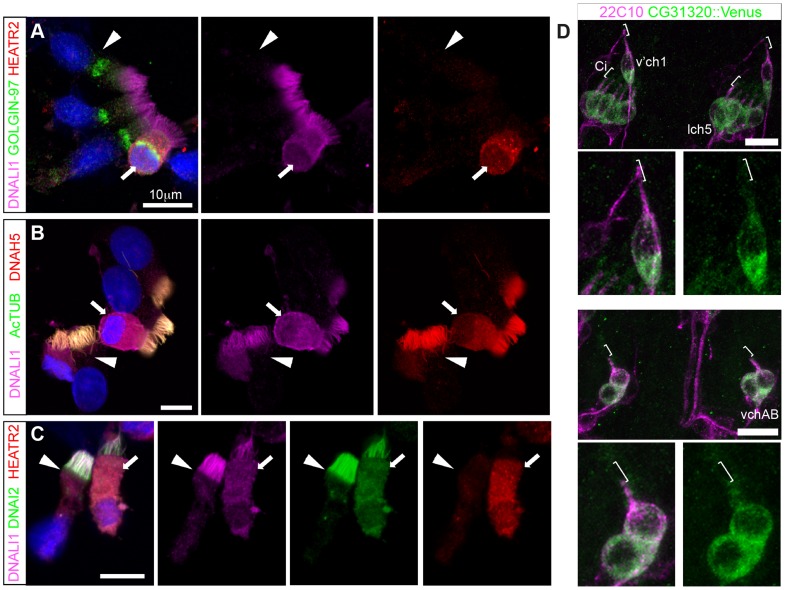
Cytoplasmic HEATR2 is expressed during early ciliogenesis. (A–C) Immunostaining of control human nasal brush epithelia reveals endogenous HEATR2 (red: Novus (A), Proteintech (C)) is highly enriched in the cytoplasm of developing MMC when components of outer dynein arms (B: DNAH5, red; C: DNAI2: green) as well as inner dynein arms (A–C: DNALI1, purple) are predominantly cytoplasmic. Arrowheads highlight fully mature MMCs where these components are exclusively axonemal and with relatively lower levels of HEATR2. Arrows highlight immature MMCs for comparison. Nuclei are stained with DAPI (blue). (Scale bar: A–C, 10 µm) (D) Double immunofluorescence of 22C10 (magenta: Futsch, cytoplasmic/membrane marker, but not cilium, of all sensory neurons) and *CG31320::mVenus* (green) indicates there is cytoplasmic but no ciliary localization of CG31320 in stage 16 Ch neurons (Ci: cilia, marked with square bracket). As this construct uses the upstream regulatory region of *CG31320* containing the X and Fox motifs to drive reporter expression, it further supports regulation occurs via these sites. (See Figure S7).

HEATR2 was an interesting candidate for functional characterization as it had not been identified in any of the ciliary axoneme [75], [76] or centrosome [44], [77] proteomic studies. This presumably was due to isolation techniques used in these studies focused on cilial/flagellar axonemes and precluded the identification of cytoplasmic components involved in ciliogenesis. Indeed, we find that endogenous HEATR2 shows granular localization throughout the cytoplasm in human nasal epithelia (Figure 8A,C), but is never detected in the ciliary axonemes even transiently in immature MMCs when the pool of outer (as represented by DNAH5 and DNAI2) and inner (as represented by DNALI1) arm dynein subunits are predominantly cytoplasmic and moving into the ciliary compartment (Figure 8A–C). This extends recent findings reported by Horani et al. [26]. Moreover, both tagged CG31320 (brackets: Figure 8D, S7) and HEATR2 (arrowheads: Figure S6G) remained cytoplasmic, without any axonemal localization, in fly Ch neurons and mouse cells respectively. Together, these data define a temporospatial window of highest HEATR2/CG31320 expression, and likely function, during early cytoplasmic dynein pre-assembly.

**Figure 9:**
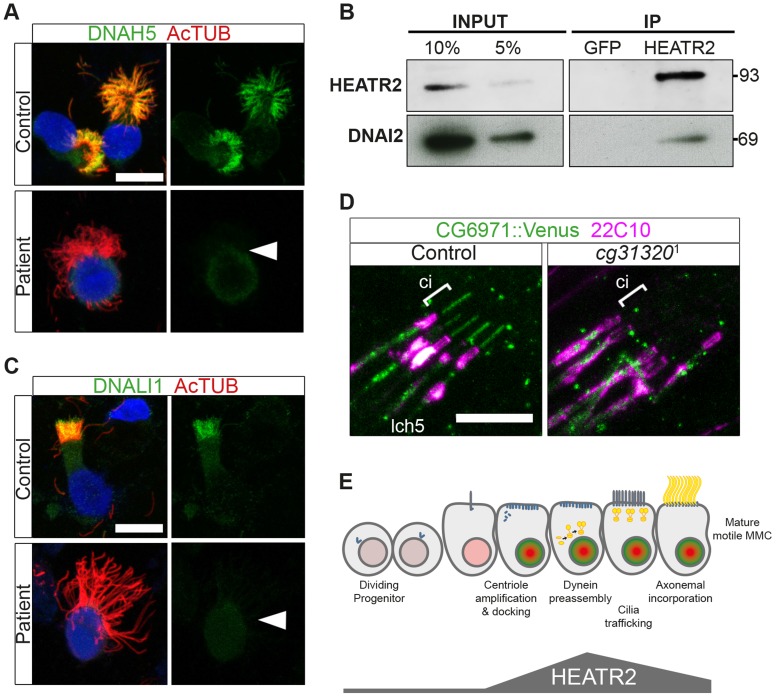
Cytoplasmic HEATR2 is required for the pre-assembly of axonemal dynein machinery necessary for motility. (A) Immunofluorescence for axonemal dynein heavy chain 5 (DNAH5: green) on respiratory cells from patients with *HEATR2* mutations compared to non-related control cells, shows loss of type 1 and type 2 DNAH5-positive staining although axonemes are still present (acetylated tubulin:red). Nuclei are stained with DAPI. (Scale bar: 10 µm). (B) Extracts prepared from control human terminally differentiated respiratory airway cultures (40 days ALI, Epithelyx) were subjected to immunoprecipitation (IP) with antibodies to HEATR2 (Proteintech) or control rabbit immunoglobulin G (GFP). Resulting immunocomplexes (IP: right) as well as dilutions of original extract (INPUT: left) were subjected to immunoblot analysis with antibodies to HEATR2 (Proteintech) or DNAI2 (Abnova). (See also Figure S8). (C) No staining of axonemal dynein light intermediate chain 1 (DNALI1: green) is observed in patients with *HEATR2* mutations. No signal above background is detected in patient cells, in contrast to strong axonemal localization in non-related control cells. Nuclei are stained with DAPI. (Scale bar: 10 µm). (D) The DNALI1 orthologue in fly, *CG6971::mVenus* (green), fails to localize to ciliary axonemes (Ci: cilia, marked with square bracket) of Ch neurons (magenta: 22C10/Futsch) in *CG31320* knock-down larvae. (E) Schematic of dynamic role of HEATR2 in developing airway epithelial MMCs. Progenitor cells exit the cell cycle to commit to the MMC lineage with primary cilia. These cells express low levels of RFX3 (light red nuclei). Other upstream factors governing multiciliogenesis (MCN, MYB) induce centriole amplification as well as expression of FOXJ1 (bright green nuclei), required for centriole docking. High FOXJ1 and RFX3 drive a cilia motility transcriptional cascade leading to high expression of HEATR2 as well as expression of axonemal dynein components. HEATR2 is involved in the pre-assembly and/or delivery of future dynein arms to the apical cilia base. In fully mature MMCs, once inner and outer arm dynein complexes are delivered and incorporated into motile ciliary axonemes, relative levels of HEATR2 as well as RFX3 and FOXJ1 are reduced. This conserved regulatory motility module is required to drive high levels of HEATR2 when axonemal dyneins are being assembled and trafficked.

Given the loss of identifiable IDA and ODA in fly *CG31320* and human *HEATR2* mutant cilia, we next asked how HEATR2 was affecting the localization of axonemal components of motile cilia. Similar to previous outer arm defects by DNAI1 immunofluorescence [26], no expression of outer arm dynein DNAH5 was detected by immunofluorescence in patient *HEATR2* mutant cilia (arrowheads: Figure 9A) [78]. Endogenous HEATR2 complexes were isolated by immunoprecipitation from terminally differentiated control human bronchial epithelial lysates and immunoblotted with antibodies against components of dynein arms (DNAH5, DNALI1, DNAI2), dynein assembly factors (KTU, DNAAF3, ZMYND10) as well as chaperones (HSP70, HSP90). In terminally differentiated cells, we were able to establish that HEATR2 interacts with DNAI2 (Figure 9B), a critical component added in the initial step of ODA assembly [79], but not other axonemal dynein components, dynein assembly factors or chaperones (Figure S8). These findings are confirmed by previous studies showing that the *Chlamydomonas* DNAI2 orthologue DIC2/IC78 is near absent in *htr2* RNAi mutant axonemal extracts [26], suggesting this interaction is conserved and of functional significance to the ODA loss phenotype in *HEATR2*-mutant cilia. Consistent with the absence of IDA from *HEATR2* PCD patient and mutant fly axonemes observed by TEM, endogenous DNALI1 was not detected in *HEATR2* PCD mutant cilia by immunofluorescence (arrowheads: Figure 9C) [80] and in *Drosophila* a tagged orthologue *CG6971::mVenus* failed to enter axonemes in *CG31320* knock-down Ch neurons (Figure 9D). The mechanism for HEATR2 in controlling IDA assembly remains unclear as we were unable to confirm interactions with DNALI1 or other DNAAFs in terminally differentiated MMCs.

Our human and fly data support a model (Figure 9E) in which HEATR2/CG31320 is a cytoplasmic factor, whose dynamic expression is elevated in an RFX- and FOX-dependent manner prior to assembly of motile cilia at the apical surface of MMCs and Ch neurons. Once mature motile cilia are assembled, HEATR2/CG31320 expression appears reduced. This corresponds to a shift from a predominantly cytoplasmic pool of precursor dynein protein subunits to assembled stable dynein complexes successfully docked on the microtubules of the axonemes. These observations suggest HEATR2 interactions are also likely very transient, as HEATR2 remains exclusively cytoplasmic throughout cilia assembly while components of dynein arms become enriched in the axonemal compartment. The interaction with DNAI2 we have identified in this study in human bronchial epithelial cultures by co-IP as well as the absence of outer dynein arms in *HEATR2/CG31320* mutant axonemes and DNAH5 localization in patient cell lines, strongly suggest that HEATR2 is functioning in the earliest steps of outer arm dynein pre-assembly and stability. This is further supported by the reports of loss of IC2 in axonemes of *htr2* mutants [26]. Whether HEATR2 also functions in apical transport of dynein arm complexes to basal bodies is unclear, as no apical inclusions of dynein arm components were observed in the loss-of-function *HEATR2* PCD cells (here and [26]). Moreover, no direct link to IFT is suggested by the fact that *Drosophila CG31320* is required for dynein arms in both IFT-dependent (Ch neurons) and IFT-independent (sperm flagella) axonemal assembly.

Although we were unable to determine how HEATR2 may be regulating inner arm dynein pre-assembly, we could show strong loss of IDA by TEM and DNALI1 IF in *HEATR2* patients and mutant flies. In contrast, algal *htr2* silenced strains showed incomplete loss of IDA by TEM and varying stability of different inner arm dynein subunits by axonemal immunoblots [26]. This suggests the role for HEATR2 may vary in the preassembly of the seven major species of inner arm dyneins in algae, each associated with an intermediate chain/light chain complex [81]. While the diversity of inner arm dyneins in modified motile cilia of other organisms is as yet poorly characterised [31], we show that the inner arm DNALI1, whose orthologue p28 is a component of several IDA species in algae, is destabilized in our PCD patients and in *Drosophila*. In contrast, Horani et al. report that inner arm dynein heavy chain DNAH7 (a component of centrin-containing inner arm dynein species b/I3′) is correctly localized in their HEATR2 PCD cilia. Consistent with this, *htr2* silenced *Chlamydomonas* strains had increased levels of centrin (component of the light chain complex of subspecies b/I3′, e/I2b and g/I3) [26]. Interestingly, while PCD patients with mutations in *DNAAF2/KTU* also show loss of light chain p28/DNALI1 immunostaining, expression levels of p28 were unchanged in the corresponding *pf13* algal mutants [30]. Changes in p28 in *htr2* silenced algal strains were not discussed [26]. Defining the currently under-appreciated molecular complexity of inner arm dyneins in functionally diverse motile cilia may help reconcile differences between PCD studies and clarify the distinct and overlapping roles for individual disease genes within the dynein cytoplasmic assembly network. Interestingly, in *CG31320* RNAi Ch axonemes, IDAs appear very cleanly absent in the majority of sections (visible IDAs 3.20% vs control 91.5%: Table 2), more so than our recent report for *ZMYND10* mutant flies (visible IDAs 21.0% vs control 86.7%) [23]. This suggests that despite overlapping gross phenotypes, important molecular subtleties with respect to IDA assembly may exist between these mutants, even within the highly modified cilium of *Drosophila* Ch neurons, which utilize a simplified ciliary motility machinery.

Our work supports a conserved role for HEATR2/CG31320 in the hierarchy of cytoplasmic factors involved in a multistep process of axonemal dynein pre-assembly In consequence, it has been agreed with the HUGO Gene Nomenclature Committee (HGNC) that *HEATR2* should be referred to as *DNAAF5*. This group includes DNAAF1/LRRC50, DNAAF2/KTU, DNAAF3/PF22, DNAAF4/DYX1C1, LRRC6, SPAG1 and ZMYND10 [22]–[31], [33]. While *Chlamydomonas* have only one outer arm dynein species comprised of a single complex of three dynein heavy chains (DHCs) with several intermediate and light chains [81], human studies suggest two types of double-headed HC ODA complexes vary in their composition along motile axonemes [78]. Unlike DNAAF2/KTU, HEATR2 is required for formation of both types of ODA subtypes whereas in *DNAAF2* mutant respiratory cilia only distal DNAH5^+^ DNAH9^+^ ODA species are affected (here and [26], [30]). Unlike ZMYND10 and LRRC6, HEATR2 is exclusively cytoplasmic [22], [23], [27], [33], [82]. Via its interaction with DNAI2, we propose HEATR2 acts in the early stages of cytoplasmic dynein preassembly, before complexes are transferred to a loading zone around the basal body where IFT comes into play. Loss of DNAI2 in *htr2* silenced algal axonemes [26] and ODA intermediate chain DNAI1 [26] and heavy chain DNAH5 (here) immunofluorescence in PCD patient cells with two different *HEATR2* mutations, strongly suggest HEATR2 may play a conserved role in stabilizing formation of the IC1/IC2 complex in the cytoplasm from the pool of precursors. Stability of *Chlamydomonas* DNAI1 (IC1) and DNAI2 (IC2) are mutually dependent, and necessary for subsequent cytoplasmic pre-assembly of outer arm heavy chains [79]. A similar role in mammalian testes has been proposed for DNAAF2/KTU [30] and the closely related PIH1D3 [83] via interaction with DNAI2. However, we have been unable to show an interaction between HEATR2 and HSP70 or HSP90 by co-IP in human mature MMCs (Figure S8A), suggesting HEATR2 is not functioning in a classical client/chaperone manner. Given no cytoplasmic accumulations of axonemal dyneins are observed in HEATR2 PCD MMCs, we instead propose the tandem arrays of HEAT repeats in HEATR2 are acting as flexible joints or scaffold stabilizing and facilitating interactions between subunits during assembly of dynein complexes.

The dynamic cytoplasmic expression of HEATR2 during the period of motile ciliogenesis suggests that its interactions may be quite transient. In the terminally differentiated human airway cultures used in our co-IP experiments, only a small fraction of cells would be in early ciliogenesis with DNAI2 cytoplasmic rather than axonemal. This would explain why only a portion of DNAI2 is co-immunoprecipitated with HEATR2 (Figure 9B). Transient interactions may be a trend for dynein assembly factors, such as PIH1D3, which localizes to the cytoplasm of spermatogenic cells but is absent from differentiated spermatids or mature sperm [83].

Intriguingly, unlike other cytoplasmic assembly PCD proteins which cause PCD when deficient, such as ZMYND10 and LRRC6 [13], [22], [23], [29], [33], this study suggests the control of *HEATR2/CG31320* expression has evolved more complexity, possibly due to independent recruitment during evolution of its HEAT repeat-dependent scaffolding functions in other non-cilia cytoplasmic assembly processes. Indeed, *CG31320* was the only gene identified in our motility candidate screen in *Drosophila* [58], [60] whose expression is not confined to Ch neurons and testes, displaying transient, yet apparently vital, mesodermal expression during embryonic development. The role of HEATR2/CG31320 outside motile ciliated cells is currently unknown. Despite its expression in non-ciliated tissues, the clinical phenotypes caused by both human *HEATR2* point mutations reported to date (here and Horani et. al 2012) only manifest in cells expressing the highest levels of HEATR2, those with motile cilia, suggesting they may be most sensitive to profoundly reduced levels of HEATR2 protein.

## Materials and Methods

### Genotyping, linkage, next generation sequencing and human mutation analysis

All patients or their parents gave informed consent and ethical approval was obtained from Leeds (East) Research Ethics Committee (07/H1306/113). DNA from three affected individuals and eleven unaffected individuals from the family was used to generate the genotype data using the Affymetrix 240K SNP arrays. Genotype data was analysed using AutoSNPa [84] and IBDfinder software [85]. A custom Agilent SureSelect pulldown reagent was used to enrich for all coding exons of the 51 UCSC-annotated genes in the locus from the genomic DNA of subject IV∶4 and sequenced the DNA using an Illumina Genome Analyzer IIx clonal sequencer. We aligned the sequence reads to the human genome (hg19) using Novoalign (Novocraft Technologies). After alignment postprocessing and variant calling using standard methods [86] only one homozygous potentially pathogenic variant remained, *ENST00000297440:c.2432-1G>C*. Presence of the mutation was confirmed by Sanger sequencing of PCR amplified products using BigDye terminator chemistry (Applied Biosystems). Primer sequences used to amplify all HEATR2 exons for sequencing are given in Table S4. DNA sequence data was analysed using the GeneScreen software [87]. The 23 additional PCD patients sequenced for *HEATR2* mutations had no known mutations at the time, and included two patients with cilia aplasia, two with absent inner dynein arms, three with absent outer dynein arms, 13 with absent inner and outer dynein arms and three with undefined defects by EM.

### LCL culture for protein and RNA extraction

To generate lymphoblastoid cell lines (LCLs), B cells from a PCD affected patient and their heterozygous parent were EBV transformed (ECACC). Two non-related, non-affected control SWEIG and FATO LCL lines were also used. LCLs were cultured in RPMI 1640 (Invitrogen), containing 10% FCS, 1 mM Oxaloacetate, 0.45 mM Pyruvate, 0.03% Glutamine, 0.2 U Insulin, 1% penicillin/streptomycin, and 8 mmol/L MOPS (pH 7.2) at 37°C, 5% CO2. Total protein extracts were made using 1× Cell Lysis Buffer (Cell Signaling Technologies) and Complete protease inhibitor tablets (Roche) and 1 mM PMSF (ThermoScientific). 20–25 µg of total protein was loaded per well of 1.0 mm 10 well pre-cast NuPage 3–8% Tris-Acetate gel (Invitrogen), according to the manufacturer's specification. Primary and secondary antibodies details are provided in Table S3. Signal was detected using ECL-Prime detection kit (GE Healthcare) and either exposed to photographic film (Kodak Biomax XAR Film) or used for digital quantification on the ImageQuant LAS 4000 (GE Healthcare) according to the manufacturer's instructions.

Total RNA was isolated according to manufacturer's protocol using RNAeasy minicolumns (Qiagen), followed by DNase treatment with Turbo DNA-free kit (Ambion). cDNA was made using First Strand Synthesis of cDNA for RT-PCR (AMV) kit (Roche). Standard RT-PCR was performed using primers spanning coding HEATR2 transcripts ENST00000297440 and predicted ENST00000313147 which uses an alternate 3′ UTR as well as final 13th exon (Table S4). Products were sent for dye terminator sequencing reactions (Applied Biosystems) on a 3130/3730 genetic analyser (Applied Biosystems). The DNA sequencing data was analysed using Sequencher 4.10.1 (Gene Codes Corp.). For quantitative analysis of splicing variants, sequencing files were analysed using the desktop program QSVanalyzer [88]. RT-qPCR was carried out on the Roche LightCycler-480 using the Roche Universal Probe Library (UPL) system. Crossing point times were identified, analysed and displayed relative to the control gene (TBP) using the Roche LC480 software. Primer and probe combinations are displayed in Table S5.

### Fly stocks

Flies were maintained on standard media at 25°C. The stocks *scaGal4*, *UAS-Dcr2* and *P{EPgy2}CG31320^EY06677^* were obtained from the Bloomington Stock Center (Indiana University, Bloomington, IN, USA). RNAi line *y w^1118^; P{KK102625}VIE-260B* and control RNAi line *y w^1118^; P{attP, y[+], w[3′]}* (landing site VIE-260B) were obtained from the Vienna RNAi Resource Centre. *BamGal4*-VP16 was obtained from H. White-Cooper. A *CG31320* deletion allele was generated by mobilizing P{EPgy2}CG31320^EY06677^ by crossing *CG31320^EY06677^* to a line containing a transposase. Screening identified a mutant line with a deletion encompassing the transcriptional start site of *CG31320*: *CG31320^27^* (992 bp deletion). Other stocks used were *rfx^49^* [35], and *fd3F^1^* [58].

### Negative geotaxis assay

A total of 20 mated female flies were placed in a measuring cylinder. After a 1 minute recovery period, the cylinder was banged firmly once on the bench, and the percentage of flies passing a 10 centimeter threshold within 1 minute of banging was recorded. This was repeated five times each for four groups of flies from each line.

### Auditory assay

For each assay, five third instar larvae were placed on an agar plate on top of a speaker. Each assay lasted 60–90 seconds, during which a 1000 Hz tone of 1 second duration was played at 30 second intervals. Larval movement was recorded by video camera and videos analysed in Macintosh iMovie software. For each tone, larval behaviour was observed in two 0.5 seconds time windows: the first starting 1 seconds before the tone, and the second starting from the onset of the tone. Number of larvae retracting (showing clear length shortening) was recorded as a score for each time window.

### Transmission electron microscopy

Whole adult fly heads were removed and rinsed in 0.5% Triton X-100. The proboscis was removed to facilitate infiltration of the fix, and the heads were then fixed in 2.5% glutaraldehyde/2% paraformaldehyde/0.1 M phosphate buffer (pH 7.4) overnight at 4°C. Heads were then washed in 0.1 M phosphate buffer (pH 7.4), postfixed with osmium tetroxide, dehydrated in an ethanol series, and embedded in Polybed812. Ultrathin (75 nm) sections of the antennae were then stained with aqueous uranyl-acetate and lead citrate and examined with a Hitachi 7000 electron microscope (Electron Microscopy Research Services, Newcastle University Medical School).

### mVenus fusion gene construction

*CG31320::mVenus* and *CG6971::mVenus* fusion genes were designed to include the upstream region containing X boxes and forkhead binding sites to allow expression from its own promoter. *CG31320* and *CG6971* were PCR amplified from genomic DNA and incorporated into pDONR221 via a BP. An LR reaction then transferred this insert into a pBID-UASC-GV vector to generate the destination vector pBID-UASC-CG31320::mVenus [89].

### Chromatin immunoprecipitation

ChIP-sequencing experiments were performed as described [90] using chromatin from either 10^7^ ependymal cultured cells from OF1 wild type mice (*O*ncins *F*rance souche *1*, Charles River Laboratory, France) [37] or mouse pancreatic cell line MIN6 [90], full datasets to be published at a later date (A.L, B.D. and W.R). Briefly ChIP was performed as described [91] using anti-RFX3 or anti-RFX1 [92]. RNAs were isolated from *Rfx3^+/+^* or *Rfx3^−/−^* mouse ependymal cells cultures prepared as previously described [37].

### Heatr2 expression studies

Due to the lack of cross-reactivity of commercially available human anti-HEATR2 antibodies, we generated custom affinity purified rabbit polyclonal antibodies against peptides from mouse Heatr2 (ENSMUSP00000026975: Dundee Cell Products). Affinity-purified antibodies against peptide 1 RETEAVVHKHRSATYC (residues 824–835 in the final exon) gave specific bands for both human HEATR2 and mouse Heatr2 by immunoblotting. This antibody is henceforth referred to as DCP49.1. Validation of human HEATR2 staining was also confirmed with several commercially available antibodies raised to different antigens: Proteintech, human FL-HEATR2 GST fusion (immunogen id# ag20080); Santa Cruz, 15–25aa peptide within 675–725aa region of human HEATR2; and Novus, 133aa peptide corresponding to 377–509 aa internal region of human HEATR2.

Animals were maintained in SPF environment and studies carried out under the guidance issued by the Medical Research Council in “Responsibility in the Use of Animals in Medical Research” (July 1993) and licensed by the Home Office under the Animals (Scientific Procedures) Act 1986. For immunofluorescence, tissues were dissected in cold phosphate buffered saline (PBS) and fixed in 4% PFA/PBS for 0.5–4 hours at 4°C with agitation. Samples were rinsed in PBS, processed through sucrose gradient for cryoprotection and embedded in OCT (Sakura). Cryostat sections of 10–15 µm were air-dried on SuperFrost plus slides (Fischer Scientific). Sections were blocked in 10% donkey serum/0.1% Triton-X in PBS and primary antibodies were diluted in 1% donkey serum/PBS (Table S3). Slides were washed and incubated in Alexafluor conjugated secondary antibodies (Table S3), washed and mounted in ProLong Gold (Life technologies).

For RT-qPCR studies, lungs and trachea were dissected from C57BL/6J embryos and neonates in cold DEPC-treated PBS into RNALater (Qiagen). Total RNA was isolated according to manufacturer's protocol using RNAeasy minicolumns with Qiashredder homogenizers (Qiagen), followed by DNase treatment with Turbo DNA-free kit (Ambion). RNA yield and A260/A280 ratio were measured using the NanoDrop ND-1000 Spectrophotometer (NanoDrop Technologies). cDNA was made using First Strand Synthesis of cDNA for RT-PCR (AMV) kit (Roche). Real-time PCR was performed on cDNA diluted 1/5 using mix in a LightCycler LC480 (Roche). Primer and probe sequences are provided in Table S5. Only a single PCR product was amplified for each reaction. To calculate relative amounts of transcripts in a sample, standard quantification curves were generated using either serial plasmid dilutions for *Tbp* and *Heatr2*, or wild-type cDNA. For each developmental time-point, three separate biological samples were isolated and processed independently. Data presented represents the mean average of these samples in three separate experimental runs with technical repeats in triplicate. Expression of each gene was normalized to reference gene *Tbp*, as it was unchanged during this developmental window and within a dynamic range of our ciliary targets. We have presented each gene relative to E14.5 (set as 1). To compare the expression level of a given gene between developmental timepoints a one-way Kruskal-Wallis analysis was used, if this generated a significant P value (<0.05) it was followed by post-hoc pairwise comparisons using Mann-Whitney U tests with Bonferroni correction for multiple testing.

### HEATR2 co-immunoprecipitation

Endogenous HEATR2 immunoprecipitations were performed using terminally differentiated normal human bronchial epithelial cells (40 days post- ALI, MucilAir, Epithelix Sarl). Whole cell lysates were prepared in lysis buffer containing 50 mM Tris-HCl pH 7.5, 100 mM NaCl, 10% Glycerol, 0.5 mM EDTA, 0.5% IGEPAL, 0.15% Triton-X 100 and Halt Protease Inhibitor Single use cocktail EDTA free (Thermo). Lysates were pre-absorbed against Dynabeads G (Invitrogen) for 30 min at 4°C to minimize non-specific binding. Pre-absorbed lysates were incubated overnight at 4°C with antibodies against HEATR2 (Proteintech) and an isotype-matched IgG rabbit polyclonal antibody (GFP: sc-8334, SantaCruz) as control. To concentrate immunocomplexes, antibody-lysate solutions were incubated with Dynabeads G followed by a series of washes with reducing amount of detergents. Finally, immunocomplexes were eluted off the beads and resolved by SDS-PAGE. Resolved immunoprecipitates were subjected to immunoblotting with antibodies to HEATR2 (Proteintech) and DNAI2 (M01, clone IC8; Abnova). Also used for immunoblotting were HSP70 (K-20; SantaCruz), (MAB32861: R&D), DNAAF2/KTU (ab99056; Abcam), DNAAF3 (ab126301; Abcam), ZMYND10/BLU (TA308345; Origene), DNAH5 (HPA037470; Sigma) and DNALI1 (N-13; SantaCruz). All primary antibody dilutions were 1/5000 and secondary antibody dilutions were 1/20000 using SuperSignal West Femto kit (Thermo) for detection.

## Supporting Information

Figure S1 *HEATR2* is mutated in human primary ciliary dyskinesia. (A) Representation of autozygosity SNP mapping on chromosome 7 generated using AutoSNPa. The scale on the left vertical is in megabases. The 3 affected individuals are on the left hand side panel, and the 11 unaffected individuals numbered are shown on the right. The black bands represent homozygous regions, and yellow bands represent heterozygous regions. The centromere is indicated by the horizontal dotted line. A 2.6 Mb region for which only the affected individuals were all concordant homozygous was identified between rs6583338 (7∶46,239) and rs7779245 (7∶3,179,991) (GRCh37). (B) Deep NGS resequencing of the 2.6 Mb interval identified a single pathogenic change as a splice acceptor mutation in the final exon of *HEATR2* (7∶766,338–829,190). The mutation (c.2432-1G>C) was homozygous in the affected individuals, and was heterozygous in the parents. It was not found in 176 ethnically matched control individuals, indicating it is was a PCD causative mutation. Sequence data was analysed using GeneScreen [87].
(TIF)

Figure S2 *HEATR2* splice mutation generates a novel and efficient +2 exonic splice acceptor to alter mRNA sequence from the final exon. (A) The *HEATR2* (*ENST00000297440:c.2432-1G>C*) mutation alters the final highly conserved consensus U2-type splice acceptor but utilizes the initial two bases of exon 13 (A–G) as an alternate and efficient splice acceptor. Mutated residue is shown in red with adjacent exonic A shown in blue. (B) RT-PCR products spanning the UK-Pakistani *HEATR2* mutation were Sanger sequenced and analysed using QSVanalyzer to quantify proportions of peaks from control and mutant transcripts [88]. The heterozygous sample was found to contain 55% of the control variant and 45% of the mutant variant. The traces also confirm the mutant transcript is efficiently spliced to the novel exonic splice acceptor, with no alternate transcripts or missplicing events visible. (C) Ribonuclease protection assay (RPA) confirms with high sensitivity and specificity splicing events in control and mutant *HEATR2* transcripts. Riboprobes containing a portion of exon 12 and 13 from a control (C) and patient (M) cDNA were generated. Sources of polyA+ RNA included a yeast control, heterozygous parent, PCD patient (affected), or an unrelated normal control. Undigested probes had a length of approximately 350 bases. Anti-sense riboprobes that annealed with identity to the transcript were digested to produce either a 245 bp control or 243 bp mutant product. Probes that annealed to sequence with a lack of identity at the exon12/13 junction were further digested to produce an exon 13 protected fragment of 163 bases in the normal or 161 bases in the mutant situation. Lack of genomic DNA contamination was confirmed using sense riboprobes. Quantification of the relative levels was 15% of the total in the patient and 24% in the control, indicating a moderate level of non-reference sequence splicing was present in both patient and control in this cell type.
(TIF)

Figure S3 *HEATR2* splice mutation results in truncation of the final conserved HEAT repeat and protein instability. (A) Schematic of the effect of the PCD transversion mutation (*ENST00000297440:c.2432-1G>C*) on translation of the protein. Based on sequencing of control, parental and patient *HEATR2* cDNA, we found the mutation results in inactivation of this splice site and utilization of the adjacent cryptic splice acceptor site in exon 13 (red box in control transcript), causing a 2-nucleotide deletion of the *HEATR2* transcript (*c.2432-2433delAG*, bases marked in red), resulting in a 2+ frameshift in translation (blue/white boxes: mutant codons). The final nucleotide of exon 12 is highlighted with an arrow, up to which point the patient sequence is the same. The mutation alters the final 44 amino acids of the protein and adds an additional 33 amino acids until it encounters a novel termination signal at codon 888 in the 3′UTR (See Figure 1B). (**B**) Western blot analysis on total protein extracts from unrelated control, parental and patient LCLs demonstrates the PCD mutation (*ENST00000297440:c.2432-1G>C*) results in a larger sized HEATR2 protein expressed at lower levels probably due to instability. Top panel left, longer exposure of blot in Figure 1E probed with anti-HEATR2 (Novus). Middle panel, same blot stripped and reprobed with anti-HEATR2 (Proteintech). Right, same samples run on a different immunoblot re-probed with anti-HEATR2 (Santa Cruz) detects the same protein bands (arrows).
(TIF)

Figure S4 *CG31320^27^* deletion results in loss of *CG31320* expression. (A) Schematic showing *CG31320^27^* deletion mutant encompassing the whole 602 bp 5′UTR as well as the ATG of *CG31320* into the first 390 bp coding sequence of the exon. (B) Comparative wild type embryo expression of *CG31320*. (C) This deletion in *CG31320^27^* mutants results in a loss of *CG31320* expression in Ch neurons.
(TIF)

Figure S5 RFX3 binds to ciliary gene promoters *in vivo*. (A) ChIP-Seq data reveals a single, specific RFX3 peak 200 bp upstream from the transcriptional start site of known Rfx target gene *Dync2li1* in OF1 mouse primary differentiated ependymal cell culture. Insert illustrates single predicted X-box within the peak sequence. (B) Well-conserved both in terms of sequence and position, a highly canonical X-box matching both Rfx-binding motifs RYYRYYN_(1–3)_ RRNRAC [42] and GTTGCCATGGCAAC [43] is identified close to the transcriptional start site of *Dync2li1*. Nucleotides are shown in grey if they vary from the consensus. (C) ChIP-Seq data reveals specific RFX3 peaks with deeper reads upstream from the transcriptional start site of both *Heatr2* and *Dync2li1* in MIN6 mouse pancreatic cell culture.
(TIF)

Figure S6 HEATR2 expression during development. (A,B) Sections of E15.5 lungs immunostained for RFX3 (red), FOXJ1 (green) and DNALI1 (purple) (A,A′) and HEATR2 (red), DNAI2 (green) and DNALI1 (purple) (B,B′). In contrast to large bore airways (see Figure 7B,C), only low nuclear levels of RFX3 are detected in small developing airways (dotted box shown in A′, B′), without FOXJ1 or target genes like DNALI1. At this stage, low levels of HEATR2 expression are observed. (C–F) Endogenous mouse HEATR2 is enriched in tissues with motile cilia including E18.5 trachea (C, C′), bronchus (D), P5 ependymal cells lining the lateral ventricles (E) and muticiliated epithelium of adult oviduct ampulla (F). (HEATR2: red, Acetylated α-tubulin: green, DAPI: blue.). (G) Over-expressed Heatr2 is cytoplasmic in ciliated murine cells. Live imaging of overexpressed mouse *Heatr2::tGFP* in murine NIH-3T3 fibroblast cells demonstrates fails to enter the primary cilia axonemes, shown by *Arl13b::mKate2*.
(TIF)

Figure S7 *CG31320::mVenus* localization during *Drosophila* development reveals cytoplasmic expression in all Ch neurons, without any cilia localization. A *CG31320::mVenus* fusion gene containing the upstream regulatory region containing X-boxes and Fox binding sites recapitulated expression from its own promoter. Double immunofluorescence with *CG31320:mVenus* (green) and structural markers (magenta) including Elav (A,B,D; nuclear, all neurons) or GT335 (D,E; polyglutamylated tubulin, cilium). (A) View of whole late stage embryo (stage 16). *CG31320::mVenus* is expressed in all Ch neurons (lch5, v'ch1, vchA, vchB in the abdominal segments). (Scale bar: 100 µm). (A′) Higher magnification view of two abdominal segments (A), showing strong cytoplasmic localization in Ch neurons (Scale bar: 20 µm). Very weak expression is also detected in v'td neuron, which although is not thought to be ciliated, remains poorly characterized and requires atonal for its development. This might be an artifact of the enhancer construct, or it might represent real expression of CG31320. (B) Higher magnification shows *CG31320::mVenus* expression is strong cytoplasmic expression in Ch neurons (Scale bar: 20 µm). Fainter, diffuse expression in one of the Ch organ support cells (scolopale cell) that ensheaths the sensory dendrite likely reflects CG31320 is expressed in the mother cell before its final division into these sister lineages. However, no clear ciliary localization of CG31320 is observed. (C) In contrast. *G6971::mVenus*, the orthologue of DNALI1 and known Fd3F target, clearly localizes to Ch neuron cilia as well as cytoplasmic staining, showing that the construct/vectors used do not interfere with cilium targeting (see also Figure 8F). (D) lch5 neurons from a third instar (mature) larva show weak *CG31320::mVenus* staining in the Ch neurons. Similar to wholemount *in-situ* staining, *CG31320* is not expressed strongly in mature neurons, being required for development. Importantly, there is no localisation to the cilia, marked by GT335. There is diffuse staining around the dendrites, again explained by some expression in the ensheathing scolopale cells. (Scale bar: 20 µm). (D′) Higher magnification view of the dendrite tips with single channel (D″), confirming no ciliary localization. (E) Expression of *CG31320::mVenus* in pupal antennal organs shows pattern restricted to mechanosensory Ch neurons, not adjacent sensory neurons highlighted by GT335 staining (Scale bar: 100 µm). (F) Localization of *CG31320::mVenus* in pupal antennal organs where cilia are stained with GT335 (Scale bar: 20 µm) shows mostly cytoplasmic staining, with some diffuse signal from ensheathing scolopale cells but no ciliary localization. (G) Schematic of Ch neurons and antibodies used for highlighting different associated structures. (H) Schematic of pupal antennal organs showing different populations of sensory neurons, with Ch neurons highlighted in green.
(TIF)

Figure S8 HEATR2 interacts with DNAI2. Protein extracts were prepared from terminally differentiated bronchial epithelial cultures from the same healthy human control and subjected to immunoprecipitation (IP) with antibodies to HEATR2 (Proteintech) or control rabbit immunoglobulin G (GFP). Resulting immunocomplexes as well as the original extracts (INPUT) were subjected to immunoblot analysis with antibodies to HEATR2, HSP70, and HSP90 (A) or DNAAF2/KTU, DNAAF3, ZMYND10 (B). Interactions with chaperones for other dynein assembly factors were not detected by HEATR2 CO-IP. (C) Blot from Figure 9B reprobed with DNAH5 (Sigma) or DNALI1 (Santa Cruz).
(TIF)

Table S1 Survey for HEATR2 orthologues across eukaryotes with presence of sensory cilia, motile cilia or flagella, as well as components of ciliary motility machinery of inner and outer dynein arms. Species examined are shown as rows, grouped as *Deuterostomia* (white), *Ecdysozoa* (yellow), *Lophotrochozoa* (orange), *Cnidaria* (purple), *Fungi* (blue), plants *and* protistan sister groups (aqua), other protist*s* (green). **Column 4**: documented presence of motile cilia/flagella. **Column 5**: Presence of sensory monocilia. **Column 6**: Expanded notes of life-cycle stages with cilia or flagella. **Column 7**: Presence and identity of orthologs in other species as described in Material and methods. Where available, an NCBI accession number is provided, alternately a reference to the relevant species database (Ensembl, JGI, Phytozome) is given. Note for *D. renio*, the presence of two putative orthologues is a scaffolding artifact due to poor genomic reference sequence within the region (≈1/3 of the gene on chromosome 3, 1/3 on an unmapped scaffold and the middle of the gene missing). **Column 8**: The similarity of HEATR2 orthologues is represented as a percentage ID with the human HEATR2 sequence from the Clustal multiple alignments. **Column 9**: The presence of absence of HEATR2 orthologues. For visual simplicity, “yes” is colored green and “no” colored red. **Columns 10–12**: Presence or absence of outer dynein arm components [93]. For visual simplicity, “yes” is colored green and “no” colored red. **Column 10**: Orthologue of heavy chain motor subunit *DNAH9* (*C.reinhardtii DHC14*)? **Column 11**: Orthologue of heavy chain motor subunit *DNAH5* (*C.reinhardtii DHC15*) ? **Column 12:** Orthologue of light chain *DNAL1* (*C.reinhardtii DLU1*)? **Columns 13–15**: Presence or absence of inner dynein arm components [93]. For visual simplicity, “yes” is colored green and “no” colored red. **Column 13**:,Orthologue of inner arm group 3 heavy chain *DNAH3* (*C. reinhardtii* gene *DHC4*, closely related to *DHC5* of the dynein species b/I3′)? **Column 14**: Orthologue of inner arm group 4 heavy chain *DNAH1* (*C.reinhardtii* gene *DHC2* of the dynein d/I2' species)? **Column 15**: Orthologue of inner arm I1/f heavy chain *DNAH2* (*C.reinhardtii* gene *DHC10*)?
(PDF)

Table S2 *CG31320/HEATR2* orthologues share conserved upstream regulatory FOX motifs and X-boxes of a master cilia motility transcriptional program. Expanded analysis of 1000 bp upstream of the *HEATR2* ATG and syntenic regions in other species to identify X box sequences, along with the nearest conserved Fox motifs. These sequences are underlined where they conform to recognized consensus sequences for generic FOX proteins (RYMAAYA (Kaufmann et al., 1995)) and RFX (RYYRYYN{1–3}RRNRAC (Laurençon et al. (2007)). Note for the identified X box site the 3′ site is extremely well-matched whilst the 5′ half-site is often more degenerate [37], [58]. A schematic version of this analysis for the closest set of Fox and X-box binding sites to the ATG is provided in Figure 6.
(PDF)

Table S3 Details of primary and secondary antibodies used in this study.
(PDF)

Table S4 Details of primers used in this study.
(PDF)

Table S5 Details of RT-qPCR primers and probes used in this study.
(PDF)
