# Supplementary figures and images for: HEATR2 Plays a Conserved Role in Assembly of the Ciliary Motile Apparatus
Source: PLoS Genet. 2014 Sep 18;10(9):e1004577. doi: 10.1371/journal.pgen.1004577 (PMC4168999; doi:10.1371/journal.pgen.1004577)

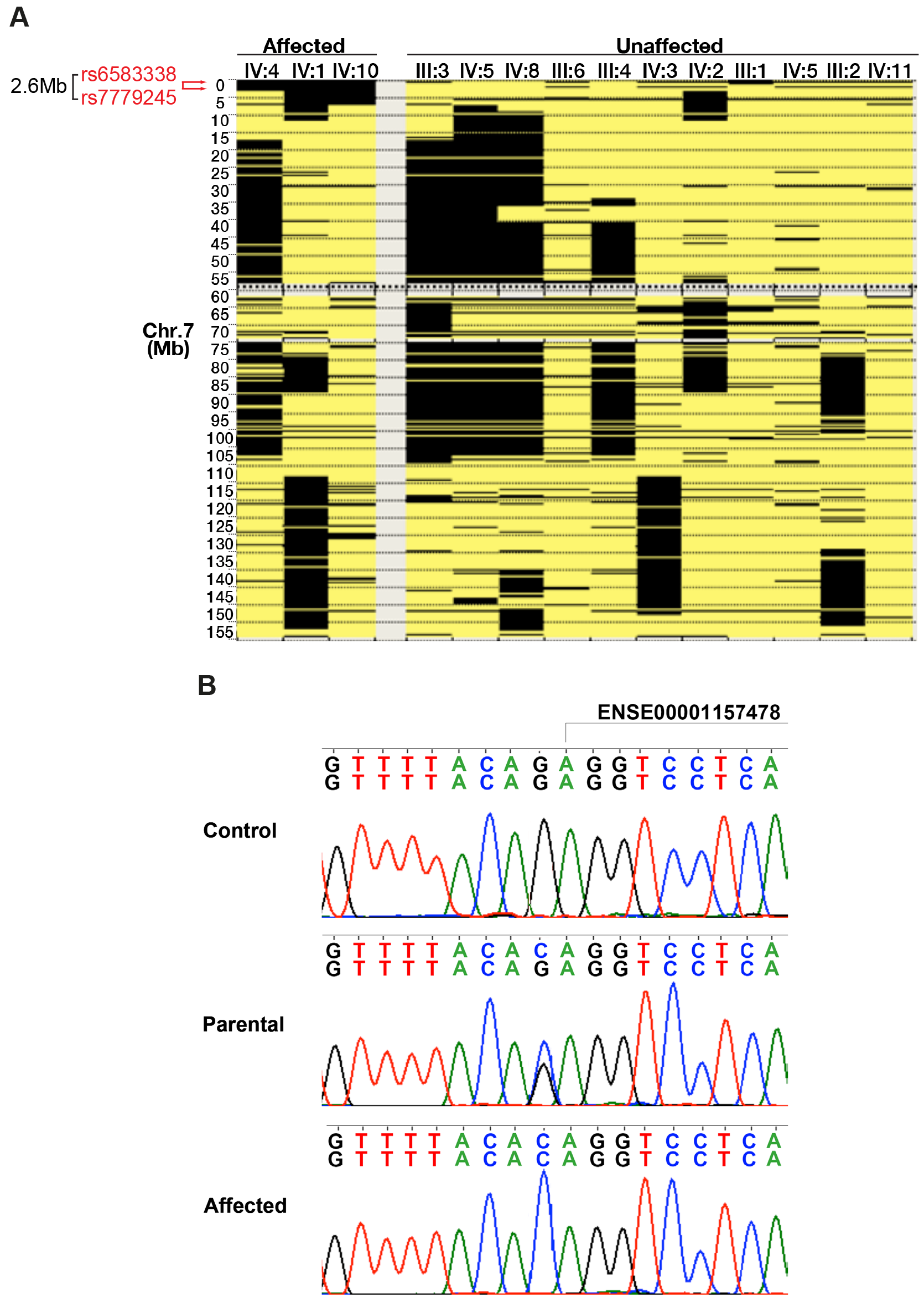

Supplement: Figure S1 — HEATR2 is mutated in human primary ciliary dyskinesia. (A) Representation of autozygosity SNP mapping on chromosome 7 generated using AutoSNPa. The scale on the left vertical is in megabases. The 3 affected individuals are on the left hand side panel, and the 11 unaffected individuals numbered are shown on the right. The black bands represent homozygous regions, and yellow bands represent heterozygous regions. The centromere is indicated by the horizontal dotted line. A 2.6 Mb region for which only the affected individuals were all concordant homozygous was identified between rs6583338 (7∶46,239) and rs7779245 (7∶3,179,991) (GRCh37). (B) Deep NGS resequencing of the 2.6 Mb interval identified a single pathogenic change as a splice acceptor mutation in the final exon of HEATR2 (7∶766,338–829,190). The mutation (c.2432-1G>C) was homozygous in the affected individuals, and was heterozygous in the parents. It was not found in 176 ethnically matched control individuals, indicating it is was a PCD causative mutation. Sequence data was analysed using GeneScreen [87]. (TIF) [file pgen.1004577.s001.tif]

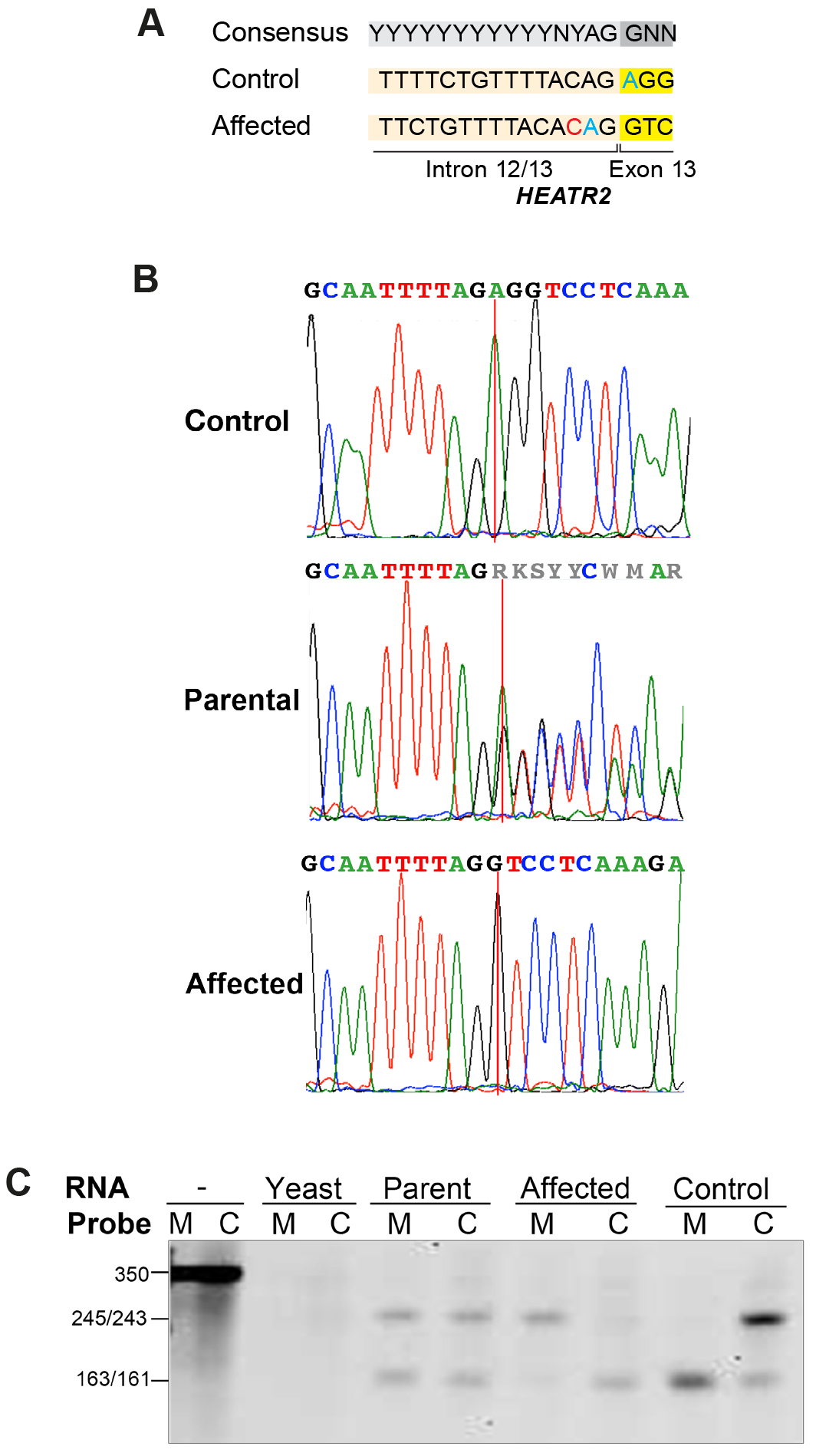

Supplement: Figure S2 — HEATR2 splice mutation generates a novel and efficient +2 exonic splice acceptor to alter mRNA sequence from the final exon. (A) The HEATR2 (ENST00000297440:c.2432-1G>C) mutation alters the final highly conserved consensus U2-type splice acceptor but utilizes the initial two bases of exon 13 (A–G) as an alternate and efficient splice acceptor. Mutated residue is shown in red with adjacent exonic A shown in blue. (B) RT-PCR products spanning the UK-Pakistani HEATR2 mutation were Sanger sequenced and analysed using QSVanalyzer to quantify proportions of peaks from control and mutant transcripts [88]. The heterozygous sample was found to contain 55% of the control variant and 45% of the mutant variant. The traces also confirm the mutant transcript is efficiently spliced to the novel exonic splice acceptor, with no alternate transcripts or missplicing events visible. (C) Ribonuclease protection assay (RPA) confirms with high sensitivity and specificity splicing events in control and mutant HEATR2 transcripts. Riboprobes containing a portion of exon 12 and 13 from a control (C) and patient (M) cDNA were generated. Sources of polyA+ RNA included a yeast control, heterozygous parent, PCD patient (affected), or an unrelated normal control. Undigested probes had a length of approximately 350 bases. Anti-sense riboprobes that annealed with identity to the transcript were digested to produce either a 245 bp control or 243 bp mutant product. Probes that annealed to sequence with a lack of identity at the exon12/13 junction were further digested to produce an exon 13 protected fragment of 163 bases in the normal or 161 bases in the mutant situation. Lack of genomic DNA contamination was confirmed using sense riboprobes. Quantification of the relative levels was 15% of the total in the patient and 24% in the control, indicating a moderate level of non-reference sequence splicing was present in both patient and control in this cell type. (TIF) [file pgen.1004577.s002.tif]

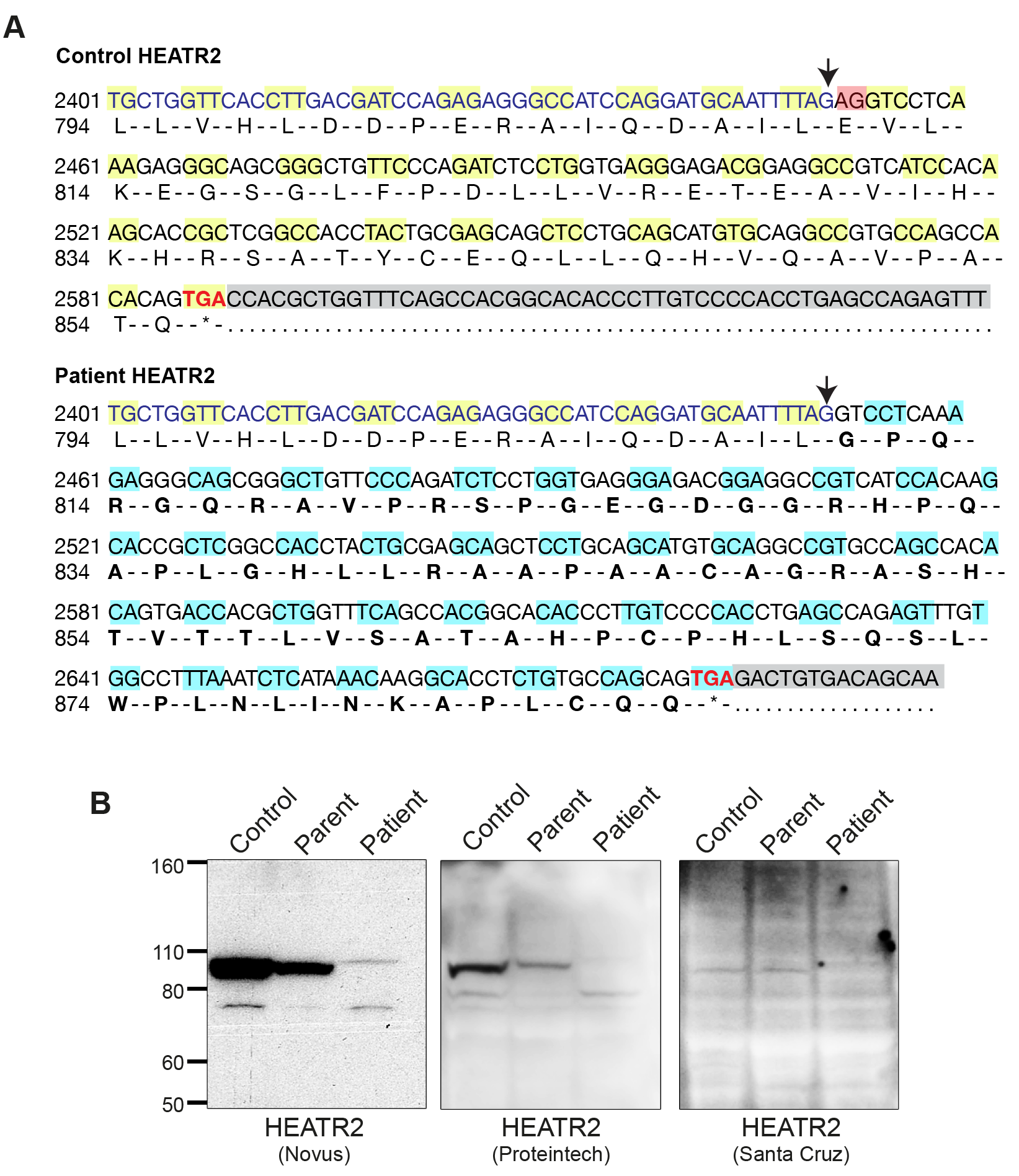

Supplement: Figure S3 — HEATR2 splice mutation results in truncation of the final conserved HEAT repeat and protein instability. (A) Schematic of the effect of the PCD transversion mutation (ENST00000297440:c.2432-1G>C) on translation of the protein. Based on sequencing of control, parental and patient HEATR2 cDNA, we found the mutation results in inactivation of this splice site and utilization of the adjacent cryptic splice acceptor site in exon 13 (red box in control transcript), causing a 2-nucleotide deletion of the HEATR2 transcript (c.2432-2433delAG, bases marked in red), resulting in a 2+ frameshift in translation (blue/white boxes: mutant codons). The final nucleotide of exon 12 is highlighted with an arrow, up to which point the patient sequence is the same. The mutation alters the final 44 amino acids of the protein and adds an additional 33 amino acids until it encounters a novel termination signal at codon 888 in the 3′UTR (See Figure 1B). (B) Western blot analysis on total protein extracts from unrelated control, parental and patient LCLs demonstrates the PCD mutation (ENST00000297440:c.2432-1G>C) results in a larger sized HEATR2 protein expressed at lower levels probably due to instability. Top panel left, longer exposure of blot in Figure 1E probed with anti-HEATR2 (Novus). Middle panel, same blot stripped and reprobed with anti-HEATR2 (Proteintech). Right, same samples run on a different immunoblot re-probed with anti-HEATR2 (Santa Cruz) detects the same protein bands (arrows). (TIF) [file pgen.1004577.s003.tif]

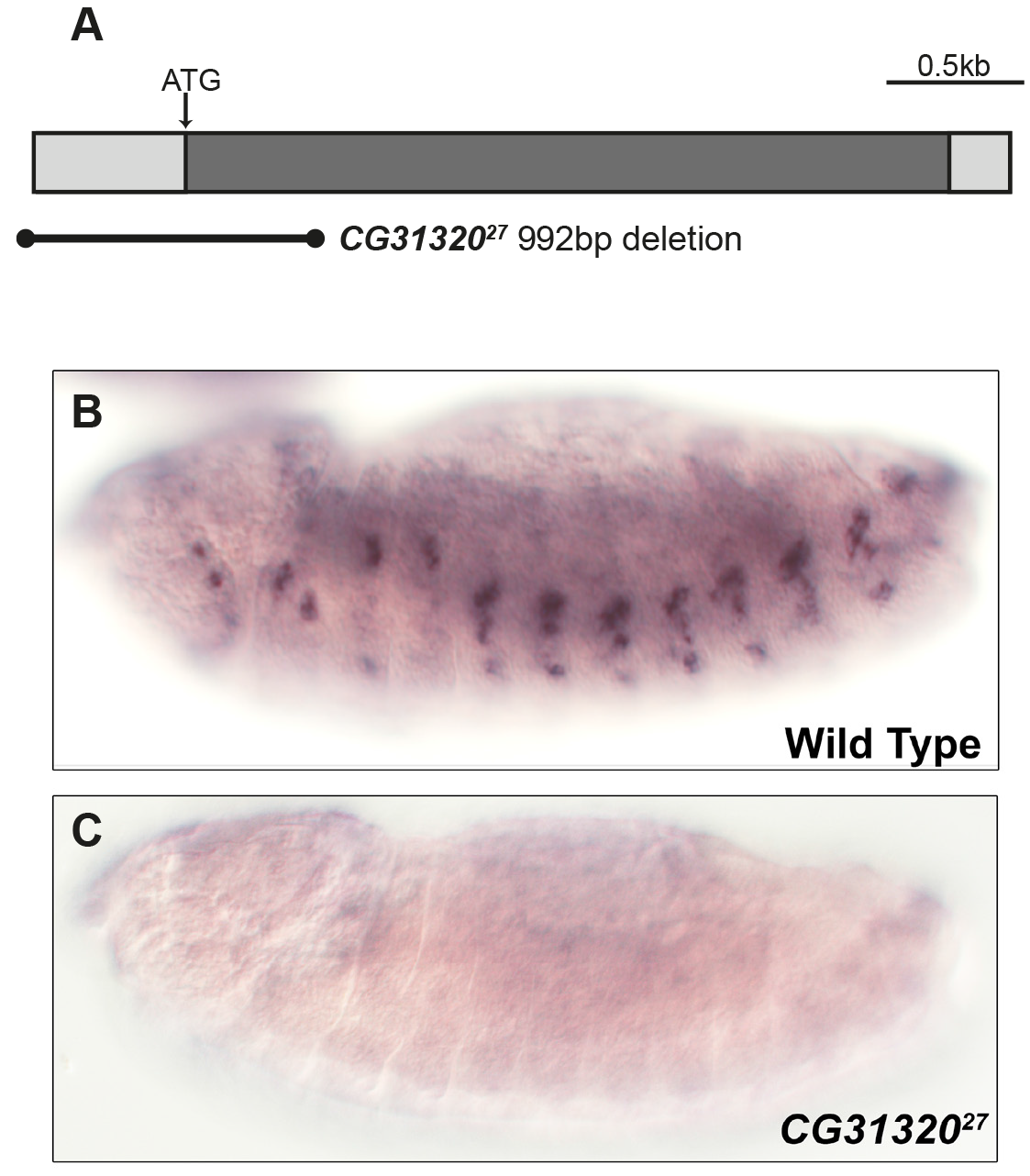

Supplement: Figure S4 — CG3132027 deletion results in loss of CG31320 expression. (A) Schematic showing CG3132027 deletion mutant encompassing the whole 602 bp 5′UTR as well as the ATG of CG31320 into the first 390 bp coding sequence of the exon. (B) Comparative wild type embryo expression of CG31320. (C) This deletion in CG3132027 mutants results in a loss of CG31320 expression in Ch neurons. (TIF) [file pgen.1004577.s004.tif]

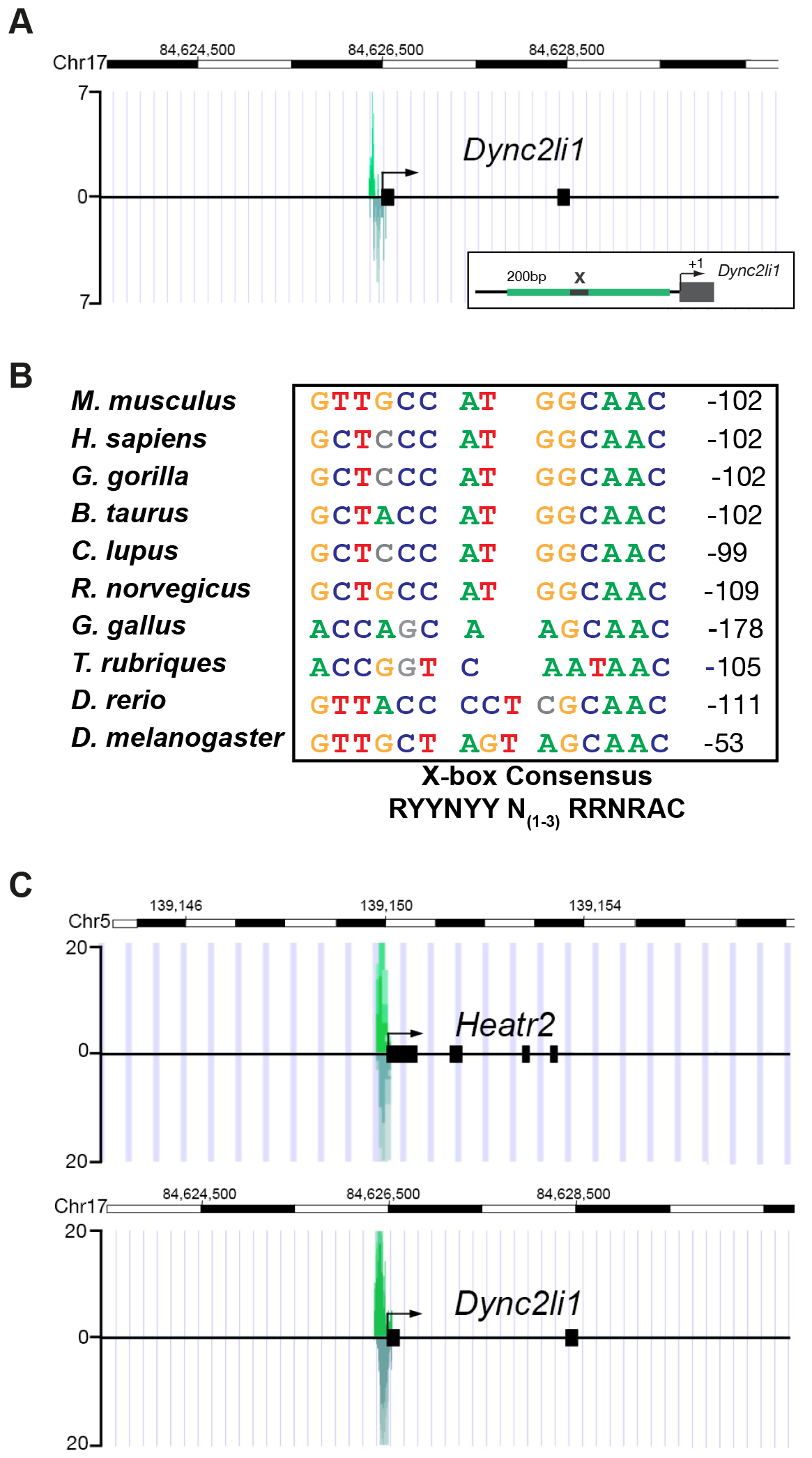

Supplement: Figure S5 — RFX3 binds to ciliary gene promoters in vivo. (A) ChIP-Seq data reveals a single, specific RFX3 peak 200 bp upstream from the transcriptional start site of known Rfx target gene Dync2li1 in OF1 mouse primary differentiated ependymal cell culture. Insert illustrates single predicted X-box within the peak sequence. (B) Well-conserved both in terms of sequence and position, a highly canonical X-box matching both Rfx-binding motifs RYYRYYN (1–3) RRNRAC [42] and GTTGCCATGGCAAC [43] is identified close to the transcriptional start site of Dync2li1. Nucleotides are shown in grey if they vary from the consensus. (C) ChIP-Seq data reveals specific RFX3 peaks with deeper reads upstream from the transcriptional start site of both Heatr2 and Dync2li1 in MIN6 mouse pancreatic cell culture. (TIF) [file pgen.1004577.s005.tif]

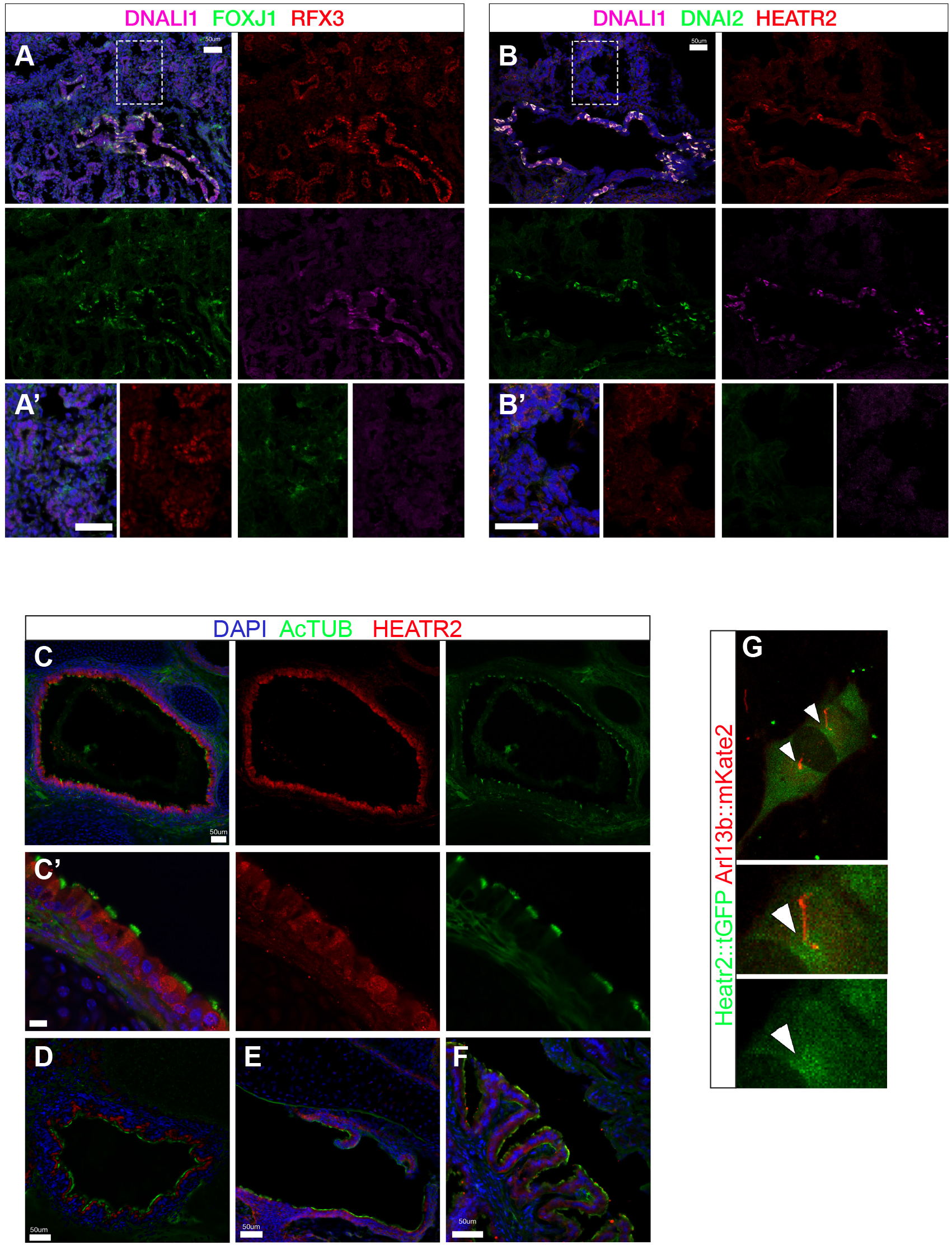

Supplement: Figure S6 — HEATR2 expression during development. (A,B) Sections of E15.5 lungs immunostained for RFX3 (red), FOXJ1 (green) and DNALI1 (purple) (A,A′) and HEATR2 (red), DNAI2 (green) and DNALI1 (purple) (B,B′). In contrast to large bore airways (see Figure 7B,C), only low nuclear levels of RFX3 are detected in small developing airways (dotted box shown in A′, B′), without FOXJ1 or target genes like DNALI1. At this stage, low levels of HEATR2 expression are observed. (C–F) Endogenous mouse HEATR2 is enriched in tissues with motile cilia including E18.5 trachea (C, C′), bronchus (D), P5 ependymal cells lining the lateral ventricles (E) and muticiliated epithelium of adult oviduct ampulla (F). (HEATR2: red, Acetylated α-tubulin: green, DAPI: blue.). (G) Over-expressed Heatr2 is cytoplasmic in ciliated murine cells. Live imaging of overexpressed mouse Heatr2::tGFP in murine NIH-3T3 fibroblast cells demonstrates fails to enter the primary cilia axonemes, shown by Arl13b::mKate2. (TIF) [file pgen.1004577.s006.tif]

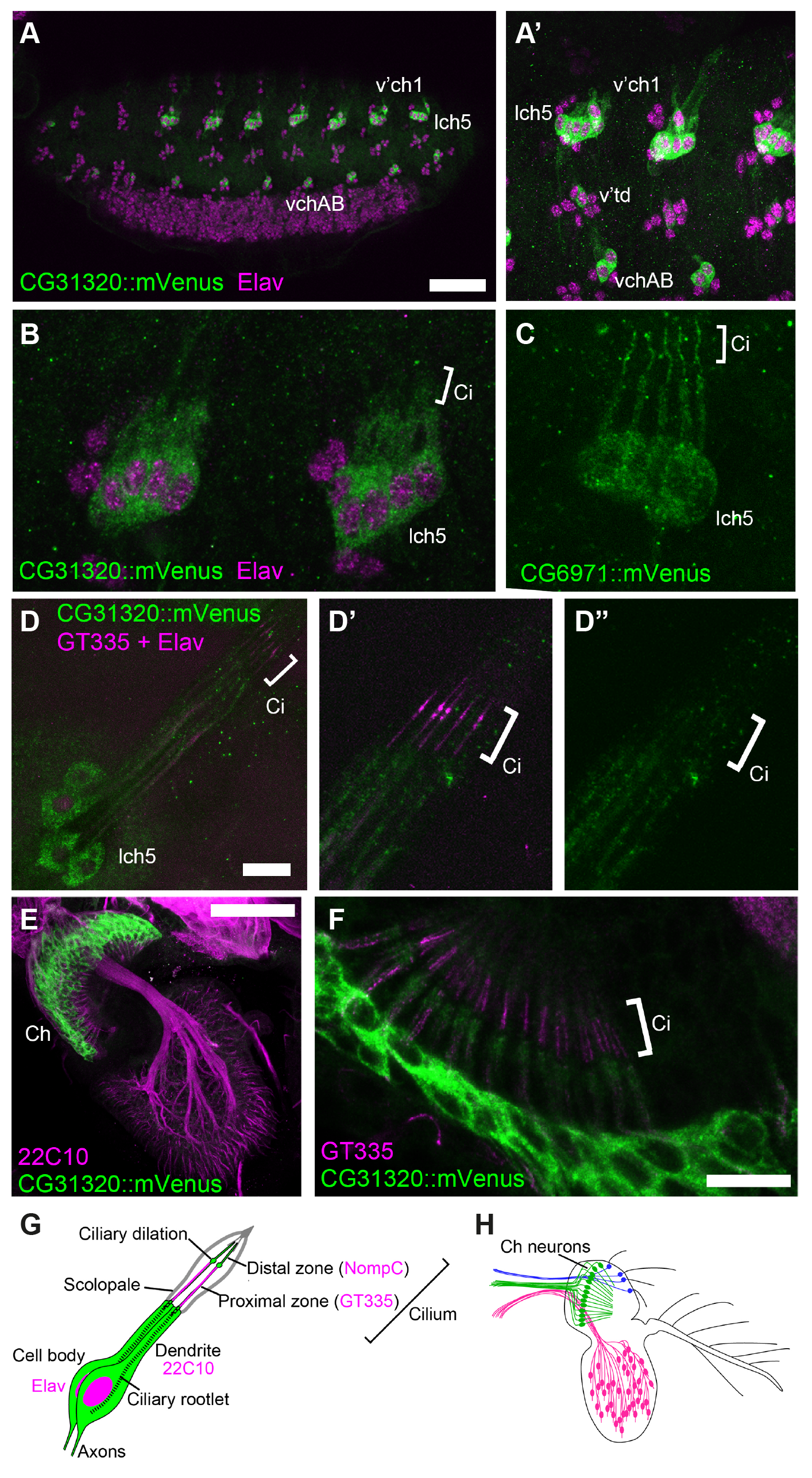

Supplement: Figure S7 — CG31320::mVenus localization during Drosophila development reveals cytoplasmic expression in all Ch neurons, without any cilia localization. A CG31320::mVenus fusion gene containing the upstream regulatory region containing X-boxes and Fox binding sites recapitulated expression from its own promoter. Double immunofluorescence with CG31320:mVenus (green) and structural markers (magenta) including Elav (A,B,D; nuclear, all neurons) or GT335 (D,E; polyglutamylated tubulin, cilium). (A) View of whole late stage embryo (stage 16). CG31320::mVenus is expressed in all Ch neurons (lch5, v'ch1, vchA, vchB in the abdominal segments). (Scale bar: 100 µm). (A′) Higher magnification view of two abdominal segments (A), showing strong cytoplasmic localization in Ch neurons (Scale bar: 20 µm). Very weak expression is also detected in v'td neuron, which although is not thought to be ciliated, remains poorly characterized and requires atonal for its development. This might be an artifact of the enhancer construct, or it might represent real expression of CG31320. (B) Higher magnification shows CG31320::mVenus expression is strong cytoplasmic expression in Ch neurons (Scale bar: 20 µm). Fainter, diffuse expression in one of the Ch organ support cells (scolopale cell) that ensheaths the sensory dendrite likely reflects CG31320 is expressed in the mother cell before its final division into these sister lineages. However, no clear ciliary localization of CG31320 is observed. (C) In contrast. G6971::mVenus, the orthologue of DNALI1 and known Fd3F target, clearly localizes to Ch neuron cilia as well as cytoplasmic staining, showing that the construct/vectors used do not interfere with cilium targeting (see also Figure 8F). (D) lch5 neurons from a third instar (mature) larva show weak CG31320::mVenus staining in the Ch neurons. Similar to wholemount in-situ staining, CG31320 is not expressed strongly in mature neurons, being required for development. Importantly, there is no localisation to [file pgen.1004577.s007.tif]

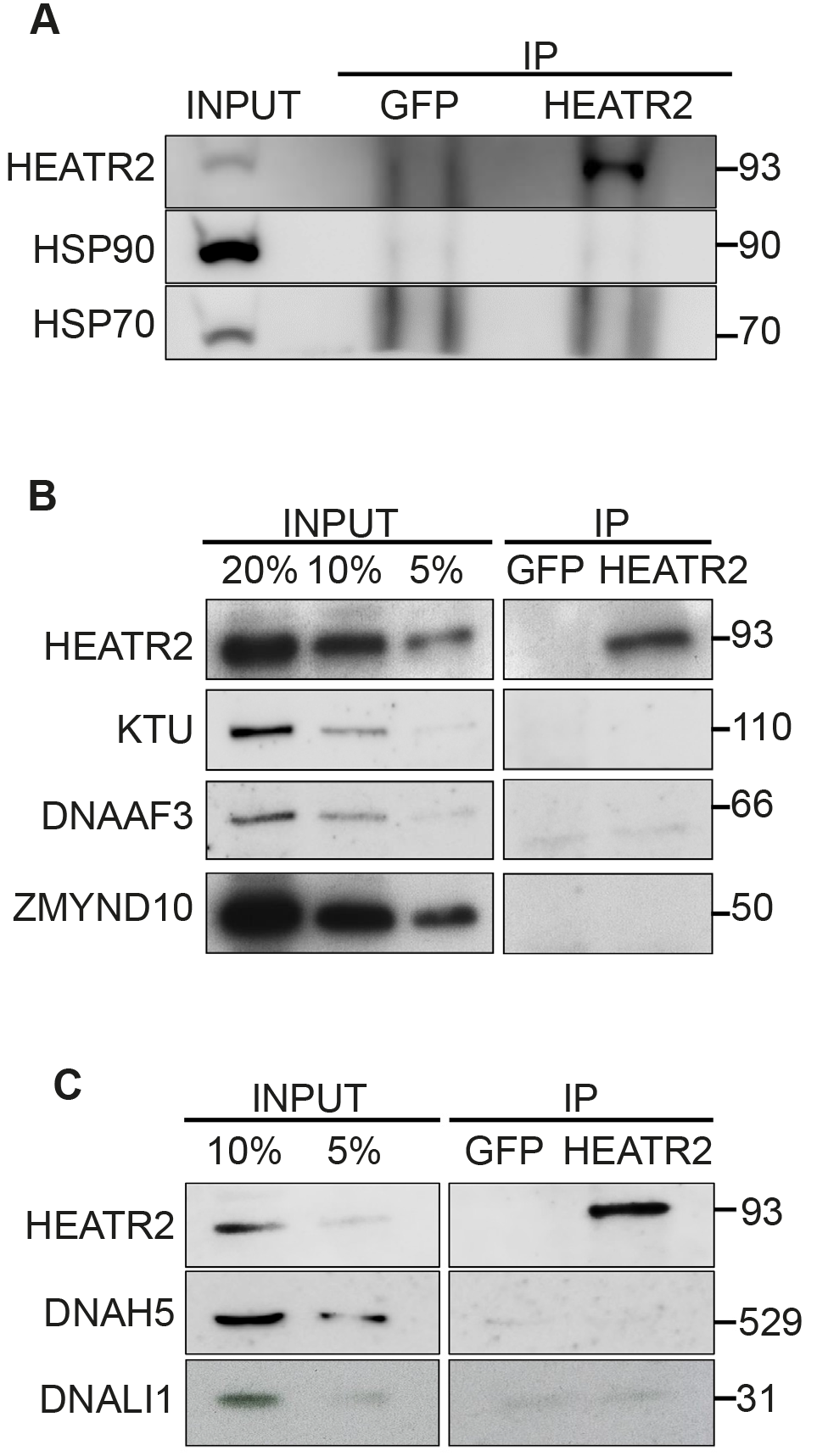

Supplement: Figure S8 — HEATR2 interacts with DNAI2. Protein extracts were prepared from terminally differentiated bronchial epithelial cultures from the same healthy human control and subjected to immunoprecipitation (IP) with antibodies to HEATR2 (Proteintech) or control rabbit immunoglobulin G (GFP). Resulting immunocomplexes as well as the original extracts (INPUT) were subjected to immunoblot analysis with antibodies to HEATR2, HSP70, and HSP90 (A) or DNAAF2/KTU, DNAAF3, ZMYND10 (B). Interactions with chaperones for other dynein assembly factors were not detected by HEATR2 CO-IP. (C) Blot from Figure 9B reprobed with DNAH5 (Sigma) or DNALI1 (Santa Cruz). (TIF) [file pgen.1004577.s008.tif]
