# Supplementary material for: HEATR2 Plays a Conserved Role in Assembly of the Ciliary Motile Apparatus
Source: PLoS Genet. 2014 Sep 18;10(9):e1004577. doi: 10.1371/journal.pgen.1004577 (PMC4168999; doi:10.1371/journal.pgen.1004577)
Supplement: Table S1 — Survey for HEATR2 orthologues across eukaryotes with presence of sensory cilia, motile cilia or flagella, as well as components of ciliary motility machinery of inner and outer dynein arms. Species examined are shown as rows, grouped as Deuterostomia (white), Ecdysozoa (yellow), Lophotrochozoa (orange), Cnidaria (purple), Fungi (blue), plants and protistan sister groups (aqua), other protists (green). Column 4: documented presence of motile cilia/flagella. Column 5: Presence of sensory monocilia. Column 6: Expanded notes of life-cycle stages with cilia or flagella. Column 7: Presence and identity of orthologs in other species as described in Material and methods. Where available, an NCBI accession number is provided, alternately a reference to the relevant species database (Ensembl, JGI, Phytozome) is given. Note for D. renio, the presence of two putative orthologues is a scaffolding artifact due to poor genomic reference sequence within the region (≈1/3 of the gene on chromosome 3, 1/3 on an unmapped scaffold and the middle of the gene missing). Column 8: The similarity of HEATR2 orthologues is represented as a percentage ID with the human HEATR2 sequence from the Clustal multiple alignments. Column 9: The presence of absence of HEATR2 orthologues. For visual simplicity, “yes” is colored green and “no” colored red. Columns 10–12: Presence or absence of outer dynein arm components [93]. For visual simplicity, “yes” is colored green and “no” colored red. Column 10: Orthologue of heavy chain motor subunit DNAH9 (C.reinhardtii DHC14)? Column 11: Orthologue of heavy chain motor subunit DNAH5 (C.reinhardtii DHC15) ? Column 12: Orthologue of light chain DNAL1 (C.reinhardtii DLU1)? Columns 13–15: Presence or absence of inner dynein arm components [93]. For visual simplicity, “yes” is colored green and “no” colored red. Column 13:,Orthologue of inner arm group 3 heavy chain DNAH3 (C. reinhardtii gene DHC4, closely related to DHC5 of the dynein species b/I3′)? Column 14: Ort [file pgen.1004577.s009.pdf]

Table S1. Survey for *HEATR2* orthologues across eukaryotes with presence of sensory cilia, motile cilia or flagella, as well as components of ciliary motility machinery of inner and outer dynein arms.

1. Species	2. Common name	3. Taxonomic Groups	4. Presence of motile cilia/flagella	5. Presence of monocilia	6. Notes	7. Orthologue accenssion no./identifier	8. Percentage ID with human HEATR2	9. presence of HEATR2	ODA 10. presence of DNAH9	ODA 11. presence of DNAH5	ODA 12. presence of DNAL1	IDA 13. presence of DNAH3	IDA 14. presence of DNAH1	IDA 15. presence of DNAH2
Homo sapiens	Human	Metazoa/Chordata/Craniata/Vertebrata/Euteleostomi/Mammalia	Y	Y		NP_060272.3		Y	Y	Y	Y	Y	Y	Y
Xenopus tropicalis	African clawed toad	Metazoa/Chordata/Craniata/Vertebrata/ Euteleostomi/Amphibia	Y	Y		XP_002941855.1	53%	Y	Y	Y	Y	Y	Y	Y
Mus musculus	Mouse	Metazoa/Chordata/Craniata/Vertebrata/Euteleostomi/Mammalia	Y	Y		NP_001074734.1	77%	Y	Y	Y	Y	Y	Y	Y
Gallus Gallus	Chicken	Metazoa/Chordata/Craniata/Vertebrata/ Euteleostomi/Archosauria/Dinosauria/Saurischia/Theropoda/ Coelurosauria/ Aves	Y	Y		XP_414755.2	56%	Y	Y	Y	Y	Y	Y	Y
Danio rerio	Zebrafish	Metazoa/Chordata/Craniata/Vertebrata/Euteleostomi/ Actinopterygii/Neopterygii /Teleostei /Ostariophysi	Y	Y		ENSDARP00000099975 & ENSDARP00000105546	43%	Y	Y	Y	Y	Y	Y	Y
Oryzias latipes	Medaka fish	Metazoa/Chordata/Craniata/ Vertebrata/Euteleostomi / Actinopterygii/Neopterygii/Teleostei/ Euteleostei	Y	Y		ENSORLP00000010519	47%	Y	Y	Y	Y	Y	N	Y
Takifugu rubripes	Puffer fish	Metazoa/Chordata/Craniata/ Vertebrata/Euteleostomi / Actinopterygii/Neopterygii/Teleostei/ Euteleostei	Y	Y		ENSTRUP00000008985	51%	Y	Y	Y	Y	Y	N	Y
Branchiostoma floridae	Amphioxus/lancelet	Metazoa/Chordata /Cephalochordata/Branchiostomidae	Y	Y	Larval movement by epidermal cilia and motile sperm	XP_002606952.1	46%	Y	Y	Y	Y	Y	Y	Y
Ciona intestinalis	Sea squirt	Metazoa/Chordata/Urochordata/Ascidiacea/ Enterogona/ Phlebobranchia/ Cionidae	Y	Y	Transient single cilia are transiently present on each ectoderm cell of the late neurula/early tailbud stage embryo, a time point just before onset of asymmetric nodal expression, posteriorly positioned, like mouse node cilia. But C. intestinalis cilia have a 9+0 ring ultrastructure and no ascribed motility. Motile sperm flagella.	XP_002127221.1	39%	Y	Y	Y	Y	Y	N	Y
Strongylocentrotus purpuratus	Purple sea urchin	Metazoa/Echinodermata/Eleutherozoa/Echinozoa/Echinoidea	Y	Y	Sea urchin embryos swim by ciliary movement as well as having a motile sperm flagella.	XP_791189.2	43%	Y	Y	Y	Y	Y	Y	Y
Drosophila melanogaster	Fruit fly	Metazoa/Arthropoda/ Hexapoda/Insecta/Pterygota/Neoptera/ Endopterygota/Diptera/ Brachycera/Muscomorpha	Y	Y* motile machinery	Chordontal organs and sperm flagella. The scolopidium, the sensillum which characterizes chordotonal organs, is an elaborate micromechanical transducer which consists of one or more bipolar sensory neurons, each with a ciliated dendrite enveloped by two specialised cells, the scolopale cell and the attachment (cap) cell, plus a glial cell.	NP_731905.1	18%	Y	Y	Y	Y	Y	N	Y
Anopheles gambiae	Mosquito	Metazoa/Arthropoda/ Hexapoda/ Insecta/ Pterygota/Neoptera/ Endopterygota/Diptera/ Nematocera	Y	Y* motile machinery	Flagellar motility and chordotonal organs.	XP_320940.5	28%	Y	Y	Y	Y	Y	N	Y (partial)
Apis mellifera	Honey bee	Metazoa/Arthropoda/Hexapoda/ Insecta/Pterygota/ Neoptera/Endopterygota/Hymenoptera/ Apocrita	Y	Y* motile machinery	Sperm flagella and chordotonal organs.	XP_393238.2	35%	Y	Y	Y	Y	Y	N	Y
Bombyx mori	Silk moth	Metazoa/Arthropoda/Hexapoda/ Insecta/ Pterygota/ Neoptera/Endopterygota/Lepidoptera	Y	Y	Motile sperm flagellla and an anomalous sensillum chaeticum with a double set of cilia and outer dendritic segments	BGIBMGA005649-TA (Ensembl)	23%	Y	Y	Y	Y	N	N	Y
Daphnia pulex	Water flea	Metazoa/ Arthropoda/Crustacea/Branchiopoda/Diplostraca	N	Y* motile machinery	Mechanosensory cilia akin to chordontal organs in *Drosophila*, although sperm are tailess or with short pseudopod extensions.	EFX78397.1	15%	Y	Y	Y	Y	N	N	N
Caenorhabditis elegans	Nematode worm	Metazoa/ Nematoda/Chromadorea/ Rhabditida	N	Y [9(1) and (2) in same cilium]		N/A	N/A	N	N	N	N	N	N	N
Brugia malayi	Filarial worm	Metazoa/Nematoda/ Chromadorea/Spirurida	N	Y		N/A	N/A	N	N	N	N	N	N	N
Capitella sp. I	Polychaete/ribbon worm	Metazoa/Annelida/ Polychaeta/ Scolecida/Capitellida	Y	Y	Fertilized eggs develop into segmented, motile metatrochophore where swimming is powered by two bands of cilia, an anterior prototroch, and a posterior telotroch. A large cluster of cilia, the neurotroch, on the ventral surface i). These ciliary bands are not only important in locomotion, but may also have a chemosensory role in settlement and metamorphosis	jgi|Capca1|167239|estExt_Genewise1Plus.C_2760033	43%	Y	Y	Y	Y	Y	Y	Y
Schistosoma mansoni	Trematode agents of schistosomiasis	Metazoa/Platyhelminthes/Trematoda/Digenea	Y	Y	Sperm flagella and a free-living miracidium stage of the platyhelminth with ciliary beat across surface.	XP_002575880.1	18%	Y	Y	Y	Y	Y	Y	Y
Helobdella robusta	Leech	Metazoa/Annelida/Clitellata/Hirudinida/ Hirudinea/ Rhynchobdellida	Y	Y	. Trochozoa produce a type of freeswimming planktonic larva, trochophore larvae that have bands of cilia in the middle.	jgi|Helro1|111152|estExt_Genewise1Plus.C_200326	35%	Y	Y	Y	Y	Y	Y	Y
Lottia gigantea	Owl limpet	Metazoa/Mollusc/Gastropoda/Patellogastropoda/Lottioidea	Y	Y	Round edge of the foot the owl limpet the pallial gill system uses currents caused by the beating of cilia to circulate water over the gills	jgi|Lotgi1|210534|estExt_Genewise1Plus.C_sca_60237	42%	Y	Y	Y	Y	Y	Y	Y
Nematostella vectensis	Star anemone	Metazoa/Cnidaria/ Anthozoa/Hexacorallia/Actiniaria/Edwardsiidae	Y	Y	Sensory structures at the apical pole of tuft of cilia in larvae: signaling functions	NEMVEDRAFT_v1g98098+genewise	37%	Y	Y	Y	Y	Y	Y	Y
Hydra magnipapillata	Hydra	Metazoa/Cnidaria/Hydrozoa/Hydroida/Anthomedusae/Hydridae	Y	?	Endodermal sensory cells had a small round exposed surface provided with a single cilium. Cnidocil at the apical end of Hydra nematocytes is a mechanosensory cilium, which acts as a ‘‘trigger’’ for discharge of the nematocyst capsule	XP_002154834.1	2.00E-148	Y	Y	Y	Y	Y	Y	Y
Trichoplax adhaerens	Placozoan	Metazoa /Placozoa	Y	?	Two cellular layers: the top epitheloid layer is made of ciliated "cover cells" flattened toward the outside of the organism, and the bottom layer is made up of cylinder cells that possess cilia used in locomotion, and gland cells that lack cilia	XP_002109889.1+ genewise	36%	Y	Y	Y	Y	Y	Y	Y
Amphimedon queenslandica	Sponge	Metazoa/Porifera/Demospongiae/Ceractinomorpha/ Haplosclerida/Niphatidae	Y	Y	Larvae have a ciliated outer epithelium. Larvae display negative phototactic behavior mediated by a ring of long-ciliated, pigmented cells at the posterior pole. Decreased expression of genes involved in assembly of cilia/flagellar structures in transition tometamorphosis may be responsible, in part, for the decrease in swimming behavior that accompanies settlement on a substrate.	XP_003385039.1	33%	Y	Y	Y	Y	Y	Y	Y
Saccharomyces cerevissiae	Baker's yeast	Fungi/Dikarya/Ascomycota/Saccharomycotina	N	N		N/A	N/A	N	N	N	N	N	N	N
Laccaria bicolor	Bicoloured deceiver fungus	Fungi/Dikarya/Basidiomycota/Agaricomycotina/ Homobasidiomycete/Agaricomycetidae/Agaricales	N	N		N/A	N/A	N	N	N	N	N	N	N
Aspergillus niger	Mold	Fungi/ Dikarya/Ascomycota/Saccharomyceta/Pezizomycotina/ Eurotiomycetes	N	N		N/A	N/A	N	N	N	N	N	N	N
Batrachochytrium dendrobatidis	Chytrid fungus infecting amphibians	Fungi/Chytridiomycota/Chytridiomycetes/Rhizophydiales/ Rhizophydiales incertae sedis	Y	N	ciliated chytrid fungus: pathogen of amphibians: have conserved IFT genes but lack most components of the BBS complex. suggests cilia were lost independently in many fungal species in evolution (common ancestor was ciliated)	jgi|Batde5|33895|estExt_fgenesh1_pg.C_10101	20%	Y	Y	Y	Y	Y	Y	Y
Populus trichocarpa	Poplar	Viridiplantae/ Streptophyta/ Embryophyta/Tracheophyta/ Euphyllophyta/Spermatophyta/Magnoliophyta/ Eudicotyledons	N	N		N/A	N/A	N	N	N	N	N	N	N

									ODA			IDA		
1. Species	2. Common name	3. Taxonomic Groups	4. Presence of motile cilia/flagella	5. Presence of monocilia	6. Notes	7. Orthologue accenssion no./identifier	8. Percentage ID with human HEATR2	9. presence of HEATR2	10. presence of DNAH9	11. presence of DNAH5	12. presence of DNAL1	13. presence of DNAH3	14. presence of DNAH1	15. presence of DNAH2
Arabidopsis thaliana	Thale cress	Viridiplantae/ Streptophyta/ Embryophyta/Tracheophyta/ Euphyllophyta/Spermatophyta/Magnoliophyta/ Eudicotyledons	N	N		N/A	N/A	N	N	N	N	N	N	N
Selaginella moellendorffii	Spikemoss/Clubmoss	Viridiplantae/Streptophyta/Embryophyta / Tracheophyta/Lycopodiophyta/Isoetopsida/Selaginellales	Yes (9+2)	N	Flagellated zoospores	XP_002985243.1	23%	Y	Y	Y	N	Y	Y	Y
Physcomitrella patens	Moss	Viridiplantae/Streptophyta/ Embryophyta/Bryophyta/Bryophytina/Bryopsida/Funariidae/ Funariales/Funariaceae	Y	N	Male gametes have flagella with motile axonemes have motile flagella without outer arm dynein.	XP_001769427.1	12%	Y	N	N	N	Y	Y	Y
Chlamydomonas reinhardtii	Unicellular alga	Viridiplantae/Chlorophyta/Chlorophyceae/ Chlamydomonadales/Chlamydomonadaceae	Y	N	Biflagellate, unicellulargreen algae	Cre09.g395500.t1.2	18%	Y	Y	Y	Y	Y	Y	Y
Volvox carteri	Simple colonial protist	Viridiplantae/Chlorophyta/Chlorophyceae/Chlamydomonadales/ Volvocaceae	Y	N	Colonial green algae of biflagellate, unicellular members.	Vocar20004522m	16%	Y	Y	Y	Y	Y	Y	Y
Chlorella variabilis NC64A	Non-motile green alga	Viridiplantae/Chlorophyta/Trebouxiophyceae/Chlorellales	?	N	Single-cell green algae, with spherical shape, assumed to be asexual and nonmotile, but the NC64A genome encodes all the known meiosis-specific proteins and a subset of proteins found in flagella. It is hypothesized that *Chlorella might* have retained a flagella-derived structure that could be involved in sexual reproduction.	jgi|ChlNC64A_1|58759|estExt_fgenesh3_pg.C_200085	12%	Y	Y	Y	Y	N	N	N
Ostreococcus lucimarinus	Sister group plant	Viridiplantae/Chlorophyta/Prasinophyceae/Mamiellales	N	N	Unicellular coccoid green alage with no flagella	N/A	N/A	N	N	N	N	N	N	Y
Micromonas sp.	Micro "alga" protist	Viridiplantae/ Chlorophyta/Prasinophyceae/Mamiellales	Y (9+2)	N	Micromonas is a eukaryotic, photosynthetic microorganism (picophytoplankton): small single-celled micro-algae. The cells are 1-3 µm long, with a single posterior flagellum divided into a very short basal part and a longer hairpoint which contains only the central pair of flagellar axoneme microtubules.	XP_002500672.1	18%	Y	Y	Y	Y	Y	Y	Y
Cyanidioschyzon merolae	Red algae	Rhodophyta/ Bangiophycea/Cyanidiales/Cyanidiaceae	N	N		N/A	N/A	N	N	N	N	N	N	N
Dictyostelium discoideum	Cellular slime mould	Amoebozoa/ Mycetozoa/Dictyosteliida	N	N	Slime mold that transitions from a collection of unicellular amoebae into a multicellular slug and then into a fruiting body . Unique asexual lifecycle that consists of four stages: vegetative, aggregation, migration, and culmination. No flagella	N/A	N/A	N	N	N	N	N	N	N
Entamoeba	Amoeba	Amoebozoa/Archamoebae/Entamoebidae	N	N	A single lobose pseudopod with a simple life cycle. The trophozoite (feeding-dividing form) is approximately 10-20 µm in diameter and feeds primarily on bacteria. It divides by simple binary fission to form two smaller daughter cells.	N/A	N/A	N	N	N	N	N	N	N
Naegleria gruberi	Amoeboflagellate	Heterolobosea/Schizopyrenida/Vahlkampfiidae	Y (9+2)	N	Free-living organism changes from an amoeba, which lacks a cytoplasmic microtubule cytoskeleton, to a flagellate, which has an elaborate microtubule cytoskeleton, including flagella. This "transformation" includes de novo synthesis of basal bodies (or centrioles).	jgi|Naegr1|56483|estExt_fgeneshHS_pg.C_10167	18%	Y	Y	Y	Y	Y	Y	Y
Monosiga brevicollis	Collar flagellate	Choanoflagellida/Codonosigidae/Monosiga	Y	N	Marine choanoflagellates: microscopic, heterotrophic single-celled and colony-forming eukaryotes with apical flagellum	XP_001747042.1	26%	Y	Y	Y	Y	Y	Y	Y
Paramecium tetraurelia	Slipper animal	Alveolata/Ciliophora/Intramacronucleata/Oligohymenophorea/ Peniculida	Y	N	Unicellular eukaryote of large size (120 micrometers) that belongs to the ciliate phylum. Ciliates share two characteristics: the presence of vibrating cilia which assure their locomotion and food capture, and nuclear dimorphism.	XP_001455888.1	7%	Y	N	N	Y	Y	Y	N
Tetrahymena thermophila	Ciliate	Alveolata/Ciliophora/Intramacronucleata/Oligohymenophorea/ Hymenostomatida	Y	N	Free-living ciliate protozoa with hundreds of motile cilia	XP_001024181.1	12%	Y	Y	Y	Y	Y	Y	Y
Plasmodium falciparum	Malaria parasite	Alveolata/ Apicomplexa/Aconoidasida/Haemosporida	Yes - no IFT genes	N	The male gamete maturation involves significant morphological development. The male gamete's DNA divides three times to form eight nuclei. Concurrently, eight flagella are formed. Each flagellum pairs with a nucleus to form a microgamete, which separates from the parasite cell. This process is referred to as exflagellation and does not require IFT.	XP_001347805.1	9%	Y	Y	Y	Y	N	Y	Y
Toxoplasma gondii	Cat toxoplasma	Alveolata/Apicomplexa/Coccidia/Eucoccidiorida/Eimeriorina/ Sarcocystidae	Yes - no IFT genes	N	parasitic protozoa: T. gondii parasitic stages lack flagella and cilia, but can move via cell crawling, but male gametes have flagella and utilize flagellar motility to reach and fertilize female gametes.	XP_002371436.1	8%	Y	Y	N	N	N	Y	Y
Thalassiosira pseudonana	Centric Diatom	Stramenopiles/Bacillariophyta/ Coscinodiscophyceae/Thalassiosirophycidae/Thalassiosirales	Yes, in sperm (9+0; no IFT-A genes	N	Marine centric diatom. Male gametes have flagella with motile axonemes without inner arm dyneins	XP_002291746.1	12%	Y	Y	Y	Y	N	N	N
Phaeodactylum tricornutum	Raphid Pennate Diatom	Stramenopiles/Bacillariophyta/Bacillariophyceae/ Bacillariophycidae/Naviculales/Phaeodactylaceae	No	N	Diatom no clear sexual reproductive state. Microalgae.	N/A	N/A	N	N	N	N	N	N	N
Aureococcus anophagefferens	Algal bloom	Stramenopiles/Pelagophyceae	Unknown. But other Pelagohyceae have flaggellated zoospores (Chrysonephos, or basal bodies Pelagococcus).	N	Spherical, non-motile very small pelagophyte which has caused destructive 'brown tide' ocean blooms. (Chrysophyte: golden algae, appears to be non-flagellate?)	N/A	N/A	N	N	N	Y	Y	Y	Y
Phytophthora sojae	Soybean pathogen	Stramenopiles/Oomycetes	y	N	Oomycete which causes a serious root and stem rot of soybeans, has motile zoospores lack cell walls and swim by means of two flagella of unequal length	EGZ28174.1	22%	Y	Y	Y	Y	Y	Y	Y
Emiliania huxleyi	Coccolithophore	Haptophyceae/Isochrysidales/Noelaerhabdaceae	Y	N	Species of photosynthetic phytoplankton with haploid (N) flagellated phase which is motile and noncalcifying.	gm1.1400361, Protein ID: 201668	N/A	Y	N	N	Y	Y	Y	N
Giardia lamblia	Flagellated protozoan parasite	Fornicata/Diplomonadida/Hexamitidae	Y		Flagellated protozoan parasite that colonizes and reproduces in the small intestine, causing giardiasis	XP_001709522.1	12%	Y	N	Y	Y	Y	Y	Y
Trypanosoma brucei brucei strain 927/4	Trypanosome	Euglenozoa/Kinetoplastida/Trypanosomatida	Y	N	Protozoan with single flagella that causes African trypanosomiasis (or sleeping sickness) in humans and nagana in animals	XP_845251.1	15%	Y	Y	Y	Y	Y	Y	Y
Trypanosoma cruzi strain CL Brener	Trypanosome	Euglenozoa/Kinetoplastida/Trypanosomatidae	Y	N	Single flagellum on parasitic trypanosome causing Chagas disease.	XP_820150.1	16%	Y	Y	Y	Y	Y	Y	Y
