# Supplementary material for: HEATR2 Plays a Conserved Role in Assembly of the Ciliary Motile Apparatus
Source: PLoS Genet. 2014 Sep 18;10(9):e1004577. doi: 10.1371/journal.pgen.1004577 (PMC4168999; doi:10.1371/journal.pgen.1004577)
Supplement: Table S2 — CG31320/HEATR2 orthologues share conserved upstream regulatory FOX motifs and X-boxes of a master cilia motility transcriptional program. Expanded analysis of 1000 bp upstream of the HEATR2 ATG and syntenic regions in other species to identify X box sequences, along with the nearest conserved Fox motifs. These sequences are underlined where they conform to recognized consensus sequences for generic FOX proteins (RYMAAYA (Kaufmann et al., 1995)) and RFX (RYYRYYN{1–3}RRNRAC (Laurençon et al. (2007)). Note for the identified X box site the 3′ site is extremely well-matched whilst the 5′ half-site is often more degenerate [37], [58]. A schematic version of this analysis for the closest set of Fox and X-box binding sites to the ATG is provided in Figure 6. (PDF) [file pgen.1004577.s010.pdf]

Table S2. *CG31320* and *HEATR2* orthologues share conserved upstream regulatory FOX motifs and X-boxes associated with a master cilia motility transcriptional program.

Organism	Name	Gene	FOX motif	Distance F to ATG	Distance F to txn	X box	Distance X to ATG	Distance between Xs	Distance F to X
D. melanogaster	***CG31320***	FBgn0051320	ATAAACA	-591	+4	GTAGCC AT AGCAAC	-536	-24	42
						GACGCT TG AGTAAC	-512		65
H. sapiens	***HEATR2***	ENSG00000164818	ACTAACA	-1036	-1016	GCTCCT TAG GATAAC	-947	-15	89
			ACCAATA	-762	-722	ACCACT ATC ATCAAC	-932		104
			TCAAACA	-379	-359	GGGGTT CC GGCGAC	-167	-158	108
			GCCTATA	-275	-235	GTTCCC CTT AGTGAC	-9		266
G. gorilla	***HEATR2***	ENSGGOG00000013100	GACAATA	-142	-142	GGGGCT CC GGCGAC	-172	-156	30
						GTTCCC CTT AGTGAC	-16		126
B. taurus	***HEATR2***	ENSBTAG00000048140	GCCAATA	-97	-97	GCAGCC A GCAGAC	-167	-151	-70
						GTCTCC CCT AGTGAC	-16		81
S. scrofa	***HEATR2***	ENSSSCG00000027809	ACCAACA	-345	-305	GTAATC CGC GCCAAC	-50	-34	280
			GCCAATA	-98	-58	GTCTCC CCT GGTGAC	-16		324
C. lupus	***HEATR2***	ENSCAFG00000011256	TCCAATA	-408	(-51)	GTCCCC GC TGCAAC	-545 (-188)		128
			GCCAATA	-97	253	GTGCTC CGC GCCAAC	-77	-61	20
						GTCTCC CCT GGCGAC	-16		81
M. musculus	***Heatr2***	ENSMUSG00000025857	GCCAATT	-237	-213	TCAGCC TCG AGCAAC	-85	-69	137
						GTTCCC TT GGAGAC	-16		-16
R. norvegicus	***Heatr2***	ENSRNOG00000021312	GCCAATA	-241	-241	GCTGCC TCG AGCAAC	-85	-69	141
						GTTCCC TT GGAGAC	-16		-16
D. ordii	***Heatr2***	ENSDORG0000000580	GTCATCA	-141	-141	GCTGTC GCG AGCGAC	-95	-79	46
						GTTCCC TT AGTGAC	-16		225
C. porcellus	***Heatr2***	ENSCPOG00000004219	CCCAACA	-211	-211	CCCGCC CC CACAAC	-178	-162	33
						GTTCCC TT GGTGAC	-16		195
G. gallus	***HEATR2***	ENSGALG00000003684	ATAAATA	-174	-174	AGCATT A AAGCAC	-126	-96	48
						GCGTCC TT AGCAAC	-30		144
X. tropicalis	***heatr2***	ENSXETG00000021643	GCAAACC	-283	-283	GTTGCC T AGCAAC	-114	-28	113
			TCCAACA	-1	-1	CCTGTT GC TGAGAC	-86		85
T. rubripes	***HEATR2***	ENSTRUG00000003816	GTCAACA	-380	-380	ACAGCC AAT CACGAC	-57	-41	5
			GCCAATC	-62	-62	GTTGTC AT AGCAAC	-16		46

RYMAAYA	RYYNYY N(1-3) RRNRAC	
	GTTGCC AT GGCAAC	
