# Supplementary material for: HEATR2 Plays a Conserved Role in Assembly of the Ciliary Motile Apparatus
Source: PLoS Genet. 2014 Sep 18;10(9):e1004577. doi: 10.1371/journal.pgen.1004577 (PMC4168999; doi:10.1371/journal.pgen.1004577)
Supplement: Table S3 — Details of primary and secondary antibodies used in this study. (PDF) [file pgen.1004577.s011.pdf]

Table S3. Details of primary and secondary antibodies used in this study.

Antigen	Antibody/Clone Name	Host Species	Dilution	Source	Application
Acetylated α-tubulin	6-11B-1	Mouse	1:1000-2000	Sigma	IF (1:500-2000)
β-actin	AC-15	Mouse	1:5000	Sigma	WB (1:5000)
CCDC39	HPA 037470	Rabbit	1:200	Sigma Atlas	WB (1:5000); IF (1:200)
DNAAF2/KTU	ab99056	Rabbit	1:5000	Abcam	WB (1:5000)
DNAAF3/C19orf51	ab126301	Rabbit	1:5000	Abcam	WB (1:5000)
DNAH5	HPA035364	Rabbit	1:200	Sigma Atlas	WB (1:5000); IF (1:200)
DNAI2	IC8	Mouse	1:100-2000	Abnova	IF (1:100), WB (1:5000)
DNALI1	HPA028305	Rabbit	1:200	Sigma Atlas	IF (1:200)
DNALI1	N-13	Goat	1:75	Santa Cruz	IF (1:75)
ELAV	mAb ELAV	Mouse	1:200	Developmental Studies Hybridoma Bank	WIHC (1:200)
FOXJ1	2A5	Mouse	1:100	LifeSpan BioSciences	IF (1:100)
Futsch	mAb-22C10	Mouse	1:200	Developmental Studies Hybridoma Bank	WIHC (1:200)
Golgin 97	CDF4	Mouse	1:250	Molecular Probes	IF ('1:250)
GFP	RbAb GFP	Rabbit	'1:500	Molecular Probes	WIHC (1:500)
HEATR2	NB1-82183	Rabbit	1:50-250	Novus Biologicals	WB (1:250); IF (1:50)
HEATR2	Proteintech	Rabbit	1:75-1000	Proteintech	WB (1:5000); IF (1:75)
HEATR2	Q-16	Goat	1:100-200	Santa Cruz	WB (1:200); IF (1:100)
Heatr2	DCP49.1	Rabbit	1:100-200	Custom	WB (1:500); IF (1:100)
HRP	RbAb-HRP	Rabbit	1:500	Jackson Laboratories	WIHC (1:500)
HSP70	K-20	Goat	1:5000	Santa Cruz	WB (1:5000)
HSP90β	MAB32861	Mouse	1:5000	R&D Systems	WB (1:5000)
NOMP C	mAb NOMP C	Mouse	1:100	Gift from J. Howard, Max Planck Institute Dresden, Germany	WIHC (1:100)
Polyglutamylated tubulin	GT335	Mouse	1:100	AdipoGen	WIHC (1:100)
RFX3	T-17	Goat	1:75	Santa Cruz	IF (1:75)
RFX3	HPA035689	Rabbit	1:100	Sigma Atlas	IF (1:100)
RFX	RbAb RFX	Rabbit	1:1000	Ref. 28	WIHC (1:1000)
ZMYND10/BLU	TA308345	Rabbit	1:5000	Origene	WB (1:5000)

Antigen	Host Species	Dilution	Source	Application
ECL α-Mouse IgG, HRP-conjugated	Sheep	1:7500-1:20000	GE Healthcare UK Ltd	WB
ECL α-Rabbit IgG, HRP-conjugated	Sheep	'1:7500-1:20000	GE Healthcare UK Ltd	WB
α-Rabbit IgG (Light Chain only) , HRP-conjugated	Mouse	1:20000	Millipore	WB
α-goat IgG-HRP	Donkey	1:10000	Santa Cruz	WB
Alexa 488, 594,647-conjugated-α-Mouse	Donkey	1:500	Molecular Probes	IF
Alexa 488, 594, 647-conjugated-α-Rabbit	Donkey	1:500	Molecular Probes	IF
Alexa 488, 594, 647-conjugated-α-Goat	Donkey	1:500	Molecular Probes	IF
