# Supplementary material for: HEATR2 Plays a Conserved Role in Assembly of the Ciliary Motile Apparatus
Source: PLoS Genet. 2014 Sep 18;10(9):e1004577. doi: 10.1371/journal.pgen.1004577 (PMC4168999; doi:10.1371/journal.pgen.1004577)
Supplement: Table S4 — Details of primers used in this study. (PDF) [file pgen.1004577.s012.pdf]

Table S4. Details of primers used in this study.

Gene	Forward Primer	Reverse Primer	Purpose
CG31320	5'-CGTTCGCTCTAAAGCCATTC-3'	5'-TCTTGTGATGCTGACGTAACCT-3'	*Drosophila* In situ hybridisation
	5'-GGGGACAAGTTTGTACAAAAAAGCAGGCTGCCGCCCAGAGTCGAACTC-3'	5'-GGGGACCACTTTGTACAAGAAAGCTGGGTCATCTTGTGATGCTGACGTAAC-3'	*Drosophila* Venus fusion reporter construct: includes 283bp upstream of 5' UTR
CG6971	5'-GGGGACAAGTTTGTACAAAAAAGCAGGCTGAATTGGAAATGTGCAACGA-3	5'-GGGGACCACTTTGTACAAGAAAGCTGGGTCCTTCTTGGGTGCGGTTATGC-3'	*Drosophila* Venus fusion reporter construct: includes 324bp upstream of 5' UTR
Fd3F	5'-AATTCCCAATTTCCGCTCTT-3'	5'-GGGCTCGAGCGCTGAAACTGGAGTCTGTTG-3'	*Drosophila* UAS misexpression construct
HEATR2	5'-GAAAGCGCTGTTCCCCTTA -3'	5'-GAGGTGGGGGTGAGAGTGT-3'	*HEATR2* gDNA sequencing (Exon 1)
	5'-GGGAGGTGAGGAACTCATAGG-3'	5'-TGGTGCATACAGCACTCTGAA-3'	*HEATR2* gDNA sequencing (Exon 2)
	5'-GTCCGTATGGCAGCACTCAG-3'	5'-GAAGCTTCCTTCAGGCTCAGA-3'	*HEATR2* gDNA sequencing (Exon 3/4)
	5'-TGTGATGTGCGGTAACTTGAG-3'	5'-GTGCACGGCTGTGTCTGG-3'	*HEATR2* gDNA sequencing (Exon 5/6)
	5'-CCCACAGACATGTCATTACGA-3'	5'-AGGGGATGCATAGGAGACAGT-3'	*HEATR2* gDNA sequencing (Exon 6)
	5'-CGACCAAATTGTACCAAGCTC-3'	5'-AGGGGATGCATAGGAGACAGT-3'	*HEATR2* gDNA sequencing (Exon 7)
	5'-GGGATAGTGAGTCCCAGCAG-3'	5'-CTTCAGTGAACCACAGCTCAG-3'	*HEATR2* gDNA sequencing (Exon 8)
	5'-ATAGTGCCCTCTCCCAGGTG-3'	5'-CAGTGCAGTGTTGGGGAAG-3'	*HEATR2* gDNA sequencing (Exon 9)
	5'-AGCCCCATTCATCCCTAGTCT-3'	5'-GAGAGCCTCTTGGGGTACG-3'	*HEATR2* gDNA sequencing (Exon 10)
	5'-TAAAGCTTTCAAGCCCCCTAA-3'	5'-ACACCCAGCCCGAGATAGTT-3'	*HEATR2* gDNA sequencing (Exon 11)
	5'-ATGCCTTTGTGGACGTTTAGA-3'	5'-TCACACGGCTCAGCTACAGAT-3'	*HEATR2* gDNA sequencing (Exon 12)
	5'-CAGCGTCAGGTTATTTGCAG-3'	5'-CACAAACTCTGGCTCAGGTG-3'	*HEATR2* gDNA sequencing (Exon 13)
	5'-AGAGCAGTGTCCAGTACCTGT-3'	5'-GTGTGCCGTGGCTGAAAC-3'	*HEATR2* RPA cloning primers (Exons 12/13)
	5'-AGTGCACCTGCTGGATCTG-3'	5'-CAAACAGTCGCTGAGCAAAA-3'	*HEATR2* cDNA RT-PCR (Exons 1-2)
	5'-AGTGATCCATTTTGGCAACG-3'	5'-GCTCATGTGGAGGGTAATGG-3'	*HEATR2* cDNA RT-PCR (Exons 2-4/5)
	5'-GTGGCAGAAGGAGAATGAGG-3'	5'-ACACCTCAGGGCTGACAAAC-3'	*HEATR2* cDNA RT-PCR (Exons 4-6)
	5'-GCCCTGAGGTGTTTCTGAAG-3'	5'-GACTGTGCGACGATGACACT-3'	*HEATR2* cDNA RT-PCR (Exons 6-8)
	5'-GCTCCTGCAGTTCAGTGTCA-3'	5'-CATCTTCGAATCCTCCTCCA-3'	*HEATR2* cDNA RT-PCR (Exons 8-11)
	5'-TCGAGACGGTGACAAAGGAC-3'	5'-ACGTGCAGGAAACACTGATG-3'	*HEATR2* cDNA RT-PCR (Exons 10/11)
	5'-ACGTGCAGGAAACACTGATG-3'	5'-TCACCAGGAGATCTGGGAAC-3'	*HEATR2* cDNA RT-PCR (Exons 11-13)
	5'-CTGGTTCACCTTGACGATCC-3'	5'-AGCCCTCTCGATGAACAGAA-3'	*HEATR2* cDNA RT-PCR (Exons 12-3'UTR)
	5'-AGTGTGTCAAGGGTGCCAAC-3'	5'-GGCCTCAGGAAACTTCCAATC-3'	*HEATR2* cDNA RT-PCR (Exons 12-3'UTR)
	5'-ACGTGCAGGAAACACTGATG-3'	5'-GGGAACCAGGCACATCTAAA-3'	*HEATR2-201* cDNA RT-PCR (Exons 11-Alternate final exon12/13)
	5'-AGTGTCATCGTCGCACAGTC-3'	5'-TCAGGGTTTGCCAAGAACTC-3'	*HEATR2* cDNA RT-PCR (Exon 8 - Intron 12/13)
GAPDH	5'-ACCACAGTCCATGCCATCAC-3'	5'-TCCACCACCCTGTTGCTGT-3'	Human *GAPDH* reference cDNA control
HPRT	5'-CAAGAGTCCTGTTGATGTG-3'	5'-GGGAAACCTCTTAGATGCTGT-3'	Human *HPRT* reference cDNA control
TBP	5'-GGGAAGGGGCATTATTTG-3'	5'-CCAGATAGCAGCACGGTA-3'	Human *TBP* reference cDNA control
