# Supplementary material for: HEATR2 Plays a Conserved Role in Assembly of the Ciliary Motile Apparatus
Source: PLoS Genet. 2014 Sep 18;10(9):e1004577. doi: 10.1371/journal.pgen.1004577 (PMC4168999; doi:10.1371/journal.pgen.1004577)
Supplement: Table S5 — Details of RT-qPCR primers and probes used in this study. (PDF) [file pgen.1004577.s013.pdf]

Table S5. Details of RT-qPCR primers and probes used in this study.

Gene Assay	Species	Protocol	Forward Primer	Reverse Primer	UPL Probe No. (Roche)
HEATR2	Human	RT-qPCR	5'-TCTGCCAGGCATCTGAAAA-3'	5'-GCCACAGTCCTCATGACACA-3'	1
TBP	Human	RT-qPCR	5'-CGGCTGTTTAACTTCGCTTC-3'	5'-CACACGCCAAGAAACAGTGA-3'	3
Bbs5	Mouse	RT-qPCR	5'-CTACCCGGTTTGAGTTTATATTC-3'	5'-CTGCACTTCTCAACTTAAAGTC-3'	n/a
Dnahc5	Mouse	RT-qPCR	5'-AAGCTGTTGCACCAGACCAT-3'	5'-CCCAGGTGGCAGTTCTGTAG-3'	88
Dnali1	Mouse	RT-qPCR	5'-AGTTCCTGAAACGGACCAAC-3'	5'-TGAGACCCATGTGGAAATGA-3'	97
Dync2li1	Mouse	RT-qPCR	5'-ACCTTAGCGTTGGAGTACAC-3'	5'-TAAGTCCAGCAAGGAGGTTC-3'	n/a
Foxj1	Mouse	RT-qPCR	5'-GAACTTCTTCCAGAACCTTCCTC-3'	5'-TCCTCCGAACACGAATGTG-3'	71
Heatr2	Mouse	RT-qPCR	5'-CTTCGATGACGTCCCTCAG-3'	5'-GTAGCGGTCCCGGAGATT-3'	46
			5'-CTCCCAACTACCCTGAGCAC-3'	5'-TGGAGAGGTTCCTGAAGACC-3'	26
			5'-TCTCAGATGCCGTGGGTCTT-3'	5'-TGCGGTAGAGGTCCTGACTG-3'	n/a
Rfx3	Mouse	RT-qPCR	5'-TGTCACCTCCAGCTGTCTCA-3'	5'-TTGAGAACCATTGCAGATTCC-3'	6
Zmynd10	Mouse	RT-qPCR	5'-GCCATCCTTGATGCAACTATC-3'	5'-CAATCAGCTCCTCCACCAG-3'	64
Dync2li1	Mouse	ChIP-qPCR	5'-ACTTCCCATGAGGCTGCTCT-3'	5'-GCAGGCTGCAAACACCTAGT-3'	n/a
Heatr2	Mouse	ChIP-qPCR	5'-CATCGTACTAGCGCCGGTTC-3'	5'-CTTCAGCCGCCTCGGTTAAG-3'	n/a
